# Molecular Clocks and Archeogenomics of a Late Period Egyptian Date Palm Leaf Reveal Introgression from Wild Relatives and Add Timestamps on the Domestication

**DOI:** 10.1093/molbev/msab188

**Published:** 2021-06-30

**Authors:** Oscar A Pérez-Escobar, Sidonie Bellot, Natalia A S Przelomska, Jonathan M Flowers, Mark Nesbitt, Philippa Ryan, Rafal M Gutaker, Muriel Gros-Balthazard, Tom Wells, Benedikt G Kuhnhäuser, Rowan Schley, Diego Bogarín, Steven Dodsworth, Rudy Diaz, Manuela Lehmann, Peter Petoe, Wolf L Eiserhardt, Michaela Preick, Michael Hofreiter, Irka Hajdas, Michael Purugganan, Alexandre Antonelli, Barbara Gravendeel, Ilia J Leitch, Maria Fernanda Torres Jimenez, Alexander S T Papadopulos, Guillaume Chomicki, Susanne S Renner, William J Baker

**Affiliations:** 1 Royal Botanic Gardens, Kew, Richmond, London, United Kingdom; 2 National Museum of Natural History, Smithsonian Institution, Washington, DC, USA; 3 Center for Genomics and Systems Biology, New York University Abu Dhabi, Abu Dhabi, United Arab Emirates; 4 French National Research Institute for Sustainable Development, Montpellier, BP, France; 5 Department of Plant Sciences, University of Oxford, Oxford, United Kingdom; 6 Lankester Botanical Garden, University of Costa Rica, San José, Costa Rica; 7 School of Biological Sciences, University of Portsmouth, Portsmouth, United Kingdom; 8 British Museum, London, United Kingdom; 9 Department of Biology, Aarhus University, Aarhus C, Denmark; 10 Institute of Biochemistry and Biology, University of Potsdam, Potsdam, Germany; 11 Department of Earth Sciences, ETH Zurich, Zurich, Switzerland; 12 Gothenburg Global Biodiversity Centre and Department of Biological and Environmental Sciences, University of Gothenburg, Gothenburg, Sweden; 13 Naturalis Biodiversity Center, Leiden, The Netherlands; 14 Molecular Ecology and Fisheries Genetics Laboratory, School of Biological Sciences, University of Bangor, Bangor, United Kingdom; 15 Department of Animal and Plant Sciences, University of Sheffield, Western Bank, Sheffield, United Kingdom; 16 Department of Biology, Washington University, Saint Louis, MO, USA

**Keywords:** ancient DNA, gene flow, population genomics, Arecaceae, archeobotany, phylogenomics

## Abstract

The date palm, *Phoenix dactylifera*, has been a cornerstone of Middle Eastern and North African agriculture for millennia. It was first domesticated in the Persian Gulf, and its evolution appears to have been influenced by gene flow from two wild relatives, *P. theophrasti*, currently restricted to Crete and Turkey, and *P. sylvestris*, widespread from Bangladesh to the West Himalayas. Genomes of ancient date palm seeds show that gene flow from *P. theophrasti* to *P. dactylifera* may have occurred by ∼2,200 years ago, but traces of *P. sylvestris* could not be detected. We here integrate archeogenomics of a ∼2,100-year-old *P. dactylifera* leaf from Saqqara (Egypt), molecular-clock dating, and coalescence approaches with population genomic tests, to probe the hybridization between the date palm and its two closest relatives and provide minimum and maximum timestamps for its reticulated evolution. The Saqqara date palm shares a close genetic affinity with North African date palm populations, and we find clear genomic admixture from both *P. theophrasti*, and *P. sylvestris*, indicating that both had contributed to the date palm genome by 2,100 years ago. Molecular-clocks placed the divergence of *P. theophrasti* from *P. dactylifera*/*P. sylvestris* and that of *P. dactylifera* from *P. sylvestris* in the Upper Miocene, but strongly supported, conflicting topologies point to older gene flow between *P. theophrasti* and *P. dactylifera*, and *P. sylvestris* and *P. dactylifera*. Our work highlights the ancient hybrid origin of the date palms, and prompts the investigation of the functional significance of genetic material introgressed from both close relatives, which in turn could prove useful for modern date palm breeding.

## Introduction

The shift from a hunter-gatherer lifestyle to a settled, agricultural subsistence strategy some 10,000–12,000 years ago was arguably one of the most important processes in human history (Diamond 2002), and together with husbandry of animals it allowed the sustained nutrition of large sedentary human population settlements (Fuller et al. 2014; Larson et al. 2014; Richter et al. 2017; Arranz-Otaegui et al. 2018). Elucidating the domestication history of major crops is thus an important scientific challenge, which requires collaboration between scholars of archeology, anthropology, taxonomy, systematics, and genomics. The widespread availability of high-throughput DNA sequencing has revolutionized the study of plant domestication history, leading to many unprecedented insights, such as the identification of crop progenitors (Ling et al. 2013; Gros-Balthazard et al. 2017; Chomicki et al. 2020; Renner et al. 2021), hybridization, and introgression events linked to the origin of crops (Cornille et al. 2012; Hufford et al. 2012; Baute et al. 2015), refinement of the geographic origins of crops (Besnard et al. 2018; Cubry et al. 2018), and the identification of genes controlling key domestication traits and more generally of convergent evolutionary processes that have challenged orthodoxies on domestication (reviewed by Allaby et al. [2019] and Purugganan [2019]). The application of genomic approaches to crop wild relatives is also bringing new resources for crop improvement (reviewed by Brozynska et al. [2016]) and food biodiversity (Przelomska et al. 2020; Borrell et al. 2020).

The date palm (*Phoenix dactylifera* L.) has been a cornerstone of Middle Eastern and North African agriculture for millennia and remains a crop of major importance with more than 9 million tonnes of fruits produced in 2019 (FAO 2021). It seems likely that wild *P. dactylifera* are native to Western Asia, although a larger historical distribution, covering all or parts of North Africa cannot be ruled out (Gros-Balthazard and Flowers 2021). Archeological evidence, ancient texts, and iconographies all point to the use of date palms for millennia in North Africa, the Middle East, and as far as Pakistan (Tengberg 2012; Gros-Balthazard and Flowers 2021; Gros-Balthazard, Baker, et al. 2021; Gros-Balthazard, Battesti, et al. 2020). The first evidence of date cultivation comes from the end of the fourth millennium B.C.E. in the Persian Gulf region (reviewed in Tengberg [2012]), and it is presumed that date palms were first domesticated in this region, perhaps from wild populations found in Oman (Gros-Balthazard et al. 2017). From the Gulf region, date palms appear to have been introduced into North Africa (Gros-Balthazard et al. 2018; Gros-Balthazard and Flowers 2021). Population-genomic analyses of date palm cultivars and wild *Phoenix* species revealed extensive introgressive hybridization of the North African date palm with *P. theophrasti* Greuter from Crete and Turkey—up to 18% of the genome of North African cultivars was shared with this species (Flowers et al. 2019), and an analysis of DNA from germinated ∼2,200-year-old seeds of *P. dactylifera* further supports this hybridization by ∼2,200 years ago (Gros-Balthazard et al. 2021).

Although it is now clear that date palm evolution in North Africa has been influenced by gene flow from *P. theophrasti*, introgression from another close wild relative, the sugar date palm *P. sylvestris*, requires further testing. Sugar date palm today occurs from Bangladesh and Southeast India to Nepal, Pakistan, and the West Himalayas, as well as in Sri Lanka and Mauritius. Although some genomic studies found distinct evidence of admixture from *P. sylvestris* and cultivated date palm (Flowers et al. 2019), others found no such evidence (Gros-Balthazard et al. 2021). *Phoenix dactylifera* and *P. sylvestris* have overlapping ranges in north-western India and Pakistan, and are known to produce fertile hybrids (Newton et al. 2013).

**Fig. 1.**
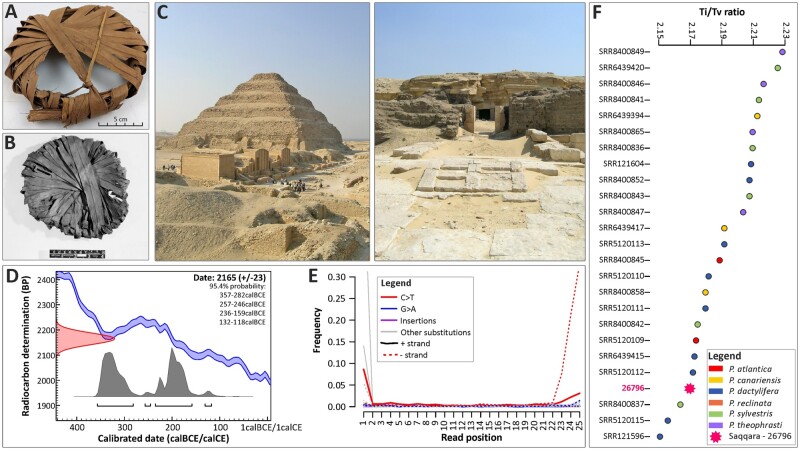
Archeological origin of the Saqqara leaf and authentication of ancient DNA. (*A*) Saqqara 26796, a jar-stopper made of date palm leaflets (excavation inventory number 102, Kew Economic Botany Collection number 26796). (*B*) A similar object to the Saqqara specimen number 26796, also made of date palm leaflets thought to be a basket-lid and found in Saqqara. (*C*) Left: Pyramid of Djoser at Saqqara, Old Kingdom; Right: Entrance to the animal tombs of the Animal necropolis of Saqqara, Late period. (*D*) Age estimation of the Saqqara date palm leaf. The distribution (red) on the *Y* axis represents the radiocarbon concentration of the Saqqara leaf as expressed in “years before present (BP),” whereas the double line line represents the known age of modern material. The gray distributions on the *X* axis indicate the likelihood of possible ages of the Saqqara leaf. (*E*) DNA misincorporations for each nucleotide position in the Saqqara date palm leaf (see supplementary fig. S1, Supplementary Material online, for detailed comparisons). (*F*) Transition/Transversion ratio of the Saqqara date palm leaf (26796) compared with modern accessions of *Phoenix*. Photos: Mark Nesbitt (*A*), The Trustees of the British Museum (*B*), Manuela Lehmann (*C* and *D*).

To elucidate the possible contributions of wild species to early domesticated date palms, we sequenced the nuclear and plastid genomes of a ∼2,100-year-old date palm leaf (fig. 1) found in Saqqara, Egypt, radiocarbon-dated to the Late Period of ancient Egypt (357–118 B.C.E.). We then used population genomic tests, molecular clocks models, and gene-flow-aware multispecies coalescence (MSC) approaches on plastid and nuclear genome-wide data sets to detect ancient gene flow and to provide a temporal framework for diversification and reticulated evolution in *Phoenix*. The results imply that the genomic ancestry of the ancient Saqqara date palm can be traced to domesticated North African *P. dactylifera* and both *P. sylvestris* and *P. theophrasti*.

## Results

### DNA Sequencing of an Archeological Date Palm Leaf from Saqqara

Our archeological sample is from an object made from date palm leaflets discovered in the temple complex of the animal necropolis of Saqqara, an Egyptian UNESCO World Heritage site located 20 km south of Cairo and adjacent to the Nile valley. The object, currently held in the Economic Botany Collection at the Royal Botanic Gardens, Kew, was recovered during the 1971-2 excavation season from the “West Dump.” The site is a mixed refuse deposit dating between 500 and 300 B.C.E. that also contained other objects such as possible “brushes” made from date palm, papyri, jar-stoppers, amulets, and other debris including seeds (Martin et al. 1981). The object consists of a plaited portion of a leaf (including leaflets and rachis) and was originally considered as a “head-pad” by excavators, however there are no analogous finds from other sites supporting this interpretation. A virtually identical object from the “West Dump” at Saqqara is held in the British Museum collections (accession EA68161) where it is identified as possibly “part of the lid of a basket.” We speculate that the object, hereafter referred to as “Saqqara leaf” (fig. 1), may instead have been part of the layering used to close and seal a vessel, similar to those found in the New Kingdom (1570–1070 B.C.E) (Hope 1977). We radiocarbon-dated the Saqqara leaf to 2,165 ± 23 BP (ETH-101122), or to a calibrated date of 357–118 B.C.E, thus confirming its burial during the Late Period or the Ptolemaic Kingdom of ancient Egypt.

We sequenced ∼30 million reads from the Saqqara leaf of which up to ∼3.5% were identified to be endogenous DNA of *P. dactylifera* (supplementary file S1 and table S1, Supplementary Material online). As expected from ssDNA libraries, nucleotide misincorporations (C to T), which are indicative of DNA damage, predominantly occurred toward both ends of the reads, and remained visible even after an uracil reduction procedure. Read length distributions were centered on 35 bp (supplementary fig. S1, Supplementary Material online), consistent with sequencing data from similarly aged material (Ramos-Madrigal et al. 2016; Scott et al. 2019; Latorre et al. 2020). To assess whether these misincorporations could affect the reliability of downstream analyses involving the Saqqara leaf, we compared the Transition/Transversion (Ti/Tv) ratio and error rate (i.e., excess of derived alleles compared with a sample that is free of misincorporations; supplementary fig. S1, Supplementary Material online) of our aDNA reads with those of modern accessions. We found that the Ti/Tv ratio and error rate of the Saqqara leaf were comparable to those derived from modern accessions (2.17 [mean = 2.192, SD = 0.022] and 0.84% [mean = 0.667%, SD = 0.17], respectively). This provides a guarantee that our phylogenetic and population genomic analyses are unlikely to be biased by misincorporations in the Saqqara sample. Although the average read depth was only ∼2×, we obtained a near-complete representation of the plastid genome of the Saqqara sample, covering 95% of the plastome of modern date palms (see Materials and Methods). We also recovered up to ∼1.5 million base pairs of the *P. dactylifera* nuclear genome (supplementary table S1, Supplementary Material online).

### Phylogenetic Placement of the Saqqara Leaf

**Fig. 2.**
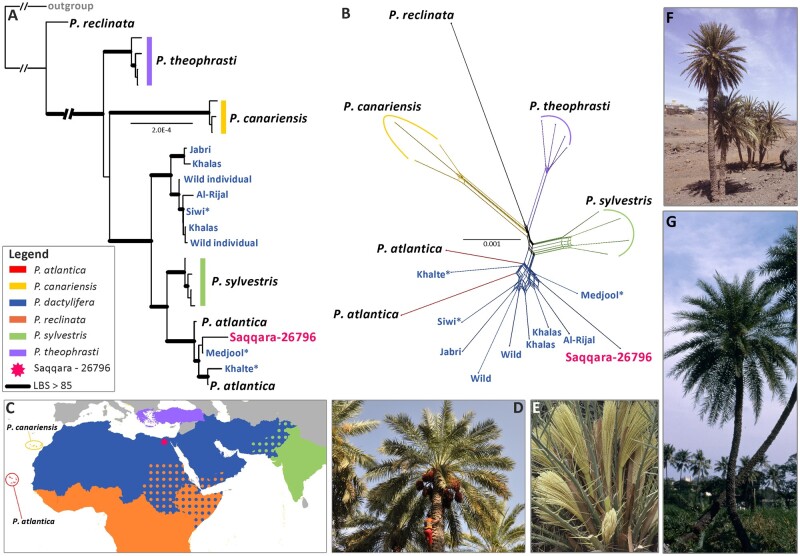
Phylogenetic placement of the Saqqara specimen amongst *Phoenix* species. (*A*) Maximum likelihood analysis of whole plastome sequences showing the placement of the Saqqara specimen amongst *Phoenix* species (accession numbers for each terminal are provided in supplementary file S2, Supplementary Material online). (*B*) Uncorrected P-distance split network produced from nuclear positions shared by the Saqqara specimen and modern accessions (Bootstrap values are provided in supplementary file S3, Supplementary Material online). North African cultivars of *P. dactylifera* are highlighted with an asterik. (*C*) Distribution map of *Phoenix dactylifera* and its closest wild relatives (the distribution ranges follow those provided by the Plants of the World Online website: http://powo.science.kew.org/). (*D*) Individual of *P. dactylifera* bearing fruits. (*E*) Male inflorescence of *P. theophrasti*. (*F*) Individuals of *P. atlantica.* (*G*) Individual of the sugar date palm (*P. sylvestris*). Photos: Penelope Dawson (*D*), John Dransfield (*E*), William J. Baker (*F*), Sasha Barrow (*G*).

To identify the closest relatives of the Saqqara leaf, we used Illumina sequencing reads available in the NCBI’s Sequence Read Archive to assemble the plastomes of 17 modern Asian and African date palms (including putative wild date palm individuals from Oman, Gros-Balthazard et al. 2017) and 17 individuals belonging to five closely related species (two *P. atlantica*, three *P. canariensis*, one *P. reclinata*, seven *P. sylvestris*, and four *P. theophrasti*; fig. 2 shows their geographic ranges and habits; supplementary table S2, Supplementary Material online). To compare the outcome of our phylogenetic and population genomic analyses with results obtained by previous studies, our taxon sampling is almost identical to Gros-Balthazard et al. (2017) and Flowers et al. (2019). Maximum likelihood (ML) phylogenetic analyses on full plastome alignments revealed that the Saqqara leaf is nested within a strongly supported clade (likelihood bootstrap support [LBS]: 100%). This group is entirely composed of North African cultivated date palms and two accessions of *P. atlantica* (fig. 2*A* and supplementary file S2, Supplementary Material online), a disputed species currently restricted to Cape Verde (Gros-Balthazard, Baker, et al. 2021) whose plastid and nuclear genetic identity are highly similar to that of North African populations of *P. dactylifera* (Gros-Balthazard *et al.* 2017; Flowers *et al.* 2019; Mohamoud *et al.* 2019). The North African clade is itself placed as sister to a clade (LBS 100%) of *P. sylvestris* samples, a species found from Bangladesh to the West Himalayas and long hypothesized to be the closest relative of *P. dactylifera* (Barrow 1998; Pintaud et al. 2013; Gros-Balthazard, Baker, et al. 2021). Asian *P. dactylifera* (LBS 100%) form a clade that is sister to the above-described clade of African *P. dactylifera* plus *P. sylvestris* (LBS 100%).

To corroborate the relationships of the Saqqara sample with modern accessions of *Phoenix*, we computed a NeighborNet split network from the same set of individuals but using a nuclear DNA alignment of sites (∼25,700 positions; supplementary table S3, Supplementary Material online) shared across modern individuals and the Saqqara sample. This approach better depicts relationships in the presence of reticulate evolution (Bogarín et al. 2018; Rutherford et al. 2018; Pérez-Escobar et al. 2020, 2021) than bifurcating trees (Bomfleur et al. 2017). The split network placed the Saqqara leaf in a group consisting of African and Asian individuals of *P. dactylifera* and *P. atlantica* with strong support (LBS 92.4%; fig. 2*B* and supplementary file S3, Supplementary Material online). The lower bootstrap support and short length of the edges connecting groups of individuals from *P. dactylifera*, *P. sylvestris*, and *P. theophrasti* suggest the existence of additional, conflicting relationships among some of the individuals that make up these species.

**Fig. 3.**
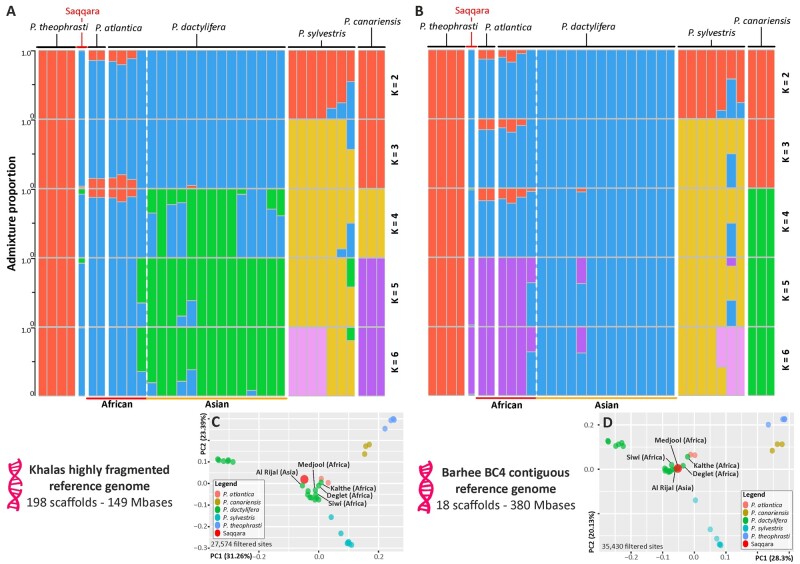
Genome ancestry of the Saqqara specimen. Population structure and principal component analyses (PCA) based on estimated nuclear GLs derived from a highly fragmented ([*A* and *C*], GCA000413155.1) and a highly contiguous reference genome ([*B* and *D*], GCA0009389715.1). Structure analyses with population number (K) from 2 to 6 (*A* and *B*) show admixture amongst wild and cultivated date palm populations, including the Saqqara leaf, and closely related *Phoenix* species. The geographical origin of modern individuals of *P. dactylifera* is provided at the bottom of the plot. Detailed cluster and delta likelihood values from K 1 to 8 are provided in supplementary figure S2, Supplementary Material online. Covariance matrices derived from PCA in (*B* and *D*) reveal a close affinity of the Saqqara specimen with modern individuals of North African *P. dactylifera* and the Cape Verde’s *P. atlantica*. The remaining individuals of *P. dactylifera* not labeled in the plots belong to Asian populations.

To test these relationships further, we determined the genomic affiliation of the nuclear genome of the Saqqara sample to either North African or Asian modern *P. dactylifera* populations. Here, we implemented a model-free principal component (PCA) and a model-based clustering analysis using genotype likelihoods derived from the nuclear genomes of all accessions, the latter assuming two to eight ancestral populations. We conducted genomic clustering analyses using as reference two different genomes of *P. dactylifera* with different levels of completeness and contiguity to account for read mapping and missing data biases (Skotte et al. 2013; Günther and Nettelblad 2019; see Materials and Methods). Regardless of the reference genome used, with four assumed ancestral populations, the Saqqara genome grouped with populations of *P. dactylifera* with African ancestry. North African individuals of *P. dactylifera* and *P. atlantica* shared most of their genome with Asian modern date palm populations albeit with a relatively small proportion of their genome admixed with *P. theophrasti* (10–15%; fig. 3 and supplementary fig. S2, Supplementary Material online), thus supporting previous findings (Flowers et al. 2019). When assuming five ancestral populations, *P. dactylifera* segregated into two populations with African and Asian ancestry, respectively (fig. 3 and supplementary fig. S2, Supplementary Material online). Most of the nuclear sequences from the Saqqara genome (90–98%) displayed components of North African domesticated *P. dactylifera* individuals and of Cape Verde’s *P. atlantica*, whilst 1–10% could be traced to both domesticated and wild Asian *P. dactylifera* individuals (fig. 3 and supplementary fig. S2, Supplementary Material online). Allele sharing between *P. dactylifera* and *P. sylvestris* was evident in clustering analyses with four and five populations, supporting previous findings by Flowers et al. (2019) but not Gros-Balthazard et al. (2021). The PCA revealed similar results to those obtained from the model-based clustering analyses. Regardless of the reference genome used, the covariance matrices inferred from 27,574 to 36,363 filtered sites placed the Saqqara date palm genome closest to modern North African date palm individuals in a cluster made of accessions of *P. dactylifera* (fig. 3*C* and *D*).

### Traces of Introgression in the Saqqara Leaf Genome

**Fig. 4.**
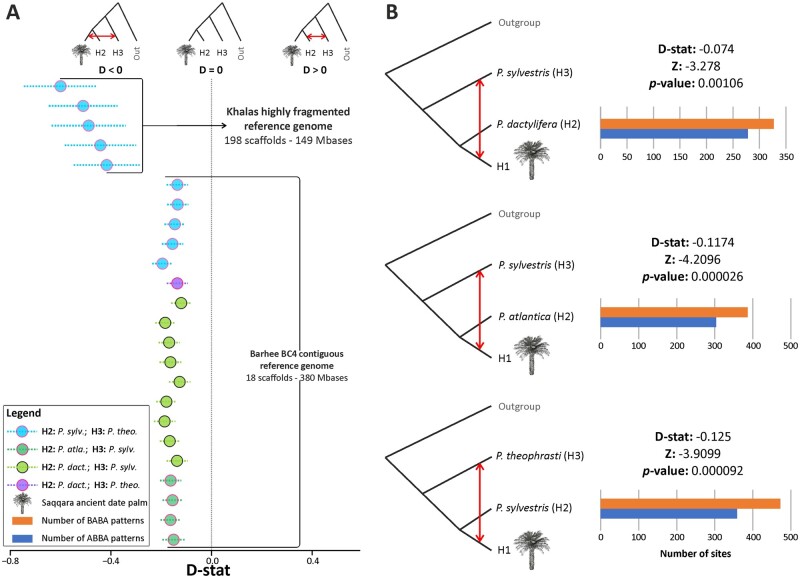
Introgression of the Saqqara leaf with modern individuals of *Phoenix sylvestris* and *P. theophrasti* inferred from nuclear bases. (*A*) Results of D-statistic analyses derived from nuclear genotype likelihoods (GLs) for the Saqqara date leaf amongst date palm (*P. dact.*) individuals and closely related species (*P. atlantica* =* P. atla.*, *P. sylvestris* =* P. sylv.*, *P. theophrasti* =* P. theo.*), with *P. reclinata* fixed as the outgroup, using as a reference the highly fragmented and contiguous reference genomes. Circles indicate the *D* value of each individual test whereas the dotted lines indicate the SD. The outcomes of all possible permutations conducted during the D-statistic test between all individuals sampled in this study are provided in supplementary tables S4 and S5 and figure S3, Supplementary Material online. (*B*) Three instances of D-statistic analyses for the Saqqara date leaf conducted amongst populations of date palms and closely related species using a contiguous reference genome supporting gene flow between *P. sylvestris* (top and mid figures), *P. theophrasti* (bottom figure) and the Saqqara date leaf. The outcomes of all possible permutations between populations and of analyses conducted using a contiguous reference genome are provided in supplementary table S6, Supplementary Material online.

To test whether alleles from *P. sylvestris*, or *P. theophrasti* could be detected in the Saqqara leaf genome, we conducted ABBA-BABA tests (i.e., D-statistics) using *P. reclinata* as an outgroup following Flowers et al. (2019) (fig. 4 and supplementary fig. S3, Supplementary Material online). To account for the differences in sequencing coverage in modern individuals compared with the ancient genome, these topological tests were conducted using two approaches tailored to separately evaluate individuals (i.e., by sampling one base from reads of one individual per population) and populations (i.e., by considering all reads from all individuals in each population; Soraggi et al. 2018). Both approaches were also implemented using two reference genomes to account for potential sequence biases (Günther and Nettelblad 2019; see Materials and Methods). Analyses considering individuals separately and populations (regardless of the genome of reference) gave similar results regarding the relatedness of the Saqqara leaf to the other taxa.

Introgression tests between modern individuals and the Saqqara leaf involved the evaluation of 1,420 and 22,292 nucleotide sites, with an average of 67 and 619 sites per analysis using highly fragmented and contiguous genome assemblies as reference, respectively (supplementary tables S4 and S5, Supplementary Material online). Although we found no signal of introgression from *P. sylvestris* in the Saqqara leaf nuclear genome using the highly fragmented genome, when instead using the contiguous genome assembly as a reference, *P. sylvestris* shared more derived alleles with the Saqqara sample than with *P. dactylifera* or *P. atlantica* (D-statistics in supplementary table S5, Supplementary Material online and fig. 4*A*). Introgression from *P. theophrasti* in the Saqqara sample was evident using both the fragmented and contiguous reference genome; when computing D (Saqqara, *P. dactylifera/P. sylvestris*; *P. theophrasti*, *P. reclinata*), *P. theophrasti* shared more derived alleles with the Saqqara sample than with *P. dactylifera* or *P. sylvestris* (*Z* < −3.26 < −4.80; fig. 4*A* and supplementary table S5, Supplementary Material online).

Population tests using the highly fragmented reference genome evaluated 2,476 to 625 bases, with an average of 432 and 125 sites per analysis considering polymorphic and nonpolymorphic sites in the outgroup, respectively (supplementary tables S6 and S7, Supplementary Material online). In contrast, analyses based on the continuous reference genome assessed 3,374 to 1,936 sites, with an average of 674 and 387 sites per analysis considering polymorphic and nonpolymorphic sites in the outgroup, respectively (supplementary tables S6 and S7, Supplementary Material online). Altogether, the results from the population-level analyses were consistent with introgression analyses conducted at the individual level, regardless of the genome of reference employed, thus providing support for the occurrence of gene flow between the Saqqara leaf, *P. theophrasti* and *P. sylvestris*. Here, when computing D (Saqqara, *P. sylvestris*; *P. theophrasti*, *P. reclinata*), *P. theophrasti* shared more derived alleles with the Saqqara leaf genome than with *P. sylvestris* (*Z* < −3.9; fig. 4*B* and supplementary tables S6 and S7, Supplementary Material online). In addition, when testing D (Saqqara, *P. atlantica/P. dactylifera*; *P. sylvestris*, *P. reclinata*), more derived alleles were shared between the Saqqara leaf genome and *P. sylvestris* than *P. sylvestris* shared with either *P. atlantica* or *P. dactylifera* (*Z* < −3.28 < −4.209; fig. 4*B* and supplementary tables S6 and S7, Supplementary Material online).

Notably, the introgression tests conducted between individuals and populations in few instances revealed positive values when computing D (Saqqara, *P. atlantica*/*P. dactylifera*; *P. theophrasti*, *P. reclinata*) (supplementary fig. S3, Supplementary Material online), thus supporting the gene flow pattern between date palms, *P atlantica* and *P. theophrasti* discovered first by Flowers et al. (2019) in North African *P. dactylifera*, but also found in the genome of ∼2,200-year-old germinated seeds of date palms from Southern Levant (Gros-Balthazard et al. 2021). Lastly, inspection of allele sharing patterns derived from analyses conducted between modern individuals and populations on the highly fragmented and contiguous reference genomes revealed widespread introgressive signals between *P. dactylifera, P. atlantica*, *P. sylvestris*, and *P. theophrasti*, all of which were statistically significant (supplementary tables S4–S7, Supplementary Material online). In particular, our finding of introgression between modern individuals *P. dactylifera* and *P. sylvestris* is in line with the study of Flowers et al. (2019), where an excess of derived allele sharing between Asian individuals of *P. dactylifera* and *P. sylvestris* was reported.

### Timing the Reticulate Evolution of *P. dactylifera* and Its Closest Wild Relatives

To obtain a time tree for *Phoenix* and further tease apart the signal of incomplete lineage sorting (ILS) from the introgressive relationships revealed by the D-statistics, we used three approaches: 1) the molecular-clock dating of plastid and nuclear genomic data sets; 2) the comparison of tree and quartet frequencies genome-wide and in scaffolds, and 3) an explicit statistical test of introgression based on ultrametric trees and DNA distance matrices. The molecular dating was performed in a framework allowing different regions of the genome to have different histories, thereby providing not only absolute ages of species divergences, but also ages for the potential gene flow events. We then quantified tree and quartet frequencies because both ILS and introgression are known to lead to nuclear intragenomic tree incongruence (Abbott et al. 2013; Pease and Hahn 2015; Gante et al. 2016). If the source of incongruence is ILS, we would generally expect a topology representing the true species relationships to occur in higher frequency, with alternative topologies to be recovered at lower and roughly equal frequencies (Gante et al. 2016). To the contrary, if tree incongruence is driven by gene flow a strong disequilibrium among the frequencies of alternative topologies is expected (Gante et al. 2016).

For the molecular-clock dating, we generated alignments of whole plastid genomes and 18 nuclear scaffolds derived from the contiguous reference genome for a set of 12 samples representing four species and the North African and Asian populations of *P. dactylifera* (supplementary table S3, Supplementary Material online). Molecular dating analyses relied on StarBEAST2, using an MSC model that enables the estimation of absolute ages in the presence of gene tree discordance (Ogilvie et al. 2017). Because the analysis is computationally intensive, we fragmented the nuclear scaffold alignments and 19 separate analyses were performed: one for each scaffold (allowing separate fragments to have different histories) and one for the plastome (representing a single linkage group).

**Fig. 5.**
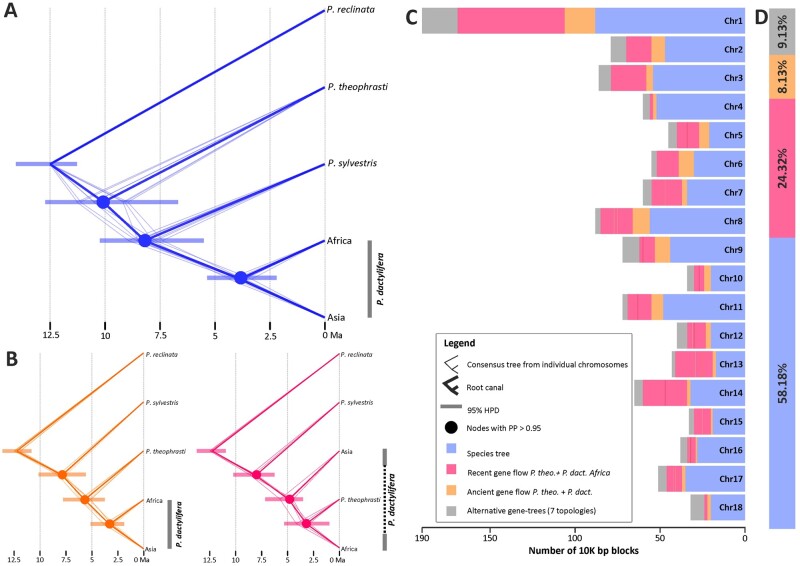
Absolute times of divergence and intragenomic tree conflict in *Phoenix*. (*A*) Chronogram of *Phoenix* reflecting the species relationships (supplementary fig. S5, Supplementary Material online). (*B*) Chronogram reflecting a closer relationship of *P. dactylifera* to *P. theophrasti* than to *P. sylvestris* (left) and of North African populations of *P. dactylifera* to *P. theophrasti* than to Asian populations of *P. dactylifera.* Thick branches represent the consensus tree as inferred from posterior distributions using the function “root canal” of DensiTree. The less thick branches represent the MCC trees derived from each nuclear scaffold. 95% Highest Posterior Density Intervals of absolute ages are provided at nodes. (*C*) Per scaffold and (*D*) genome-wide topology frequencies in *Phoenix*.

The plastid phylogeny showed North African individuals of *P. dactylifera* (including *P. atlantica*) and *P. sylvestris* as sisters to each other (posterior probability [PP]=0.86) with a mean age of divergence of ∼ 2 My (supplementary fig. S4 and table S8, Supplementary Material online). The Most Recent Common Ancestor (MRCA) of the group including Asian individuals of *P. dactylifera*, *P. sylvestris*, and North African *P. dactylifera* (PP=0.87) was estimated to be ∼3.8 My old, whereas the divergence of *P. theophrasti* from *P. dactylifera* and *P. sylvestris* (PP=0.97) was inferred to have occurred ∼7 Ma (supplementary table S8, Supplementary Material online). In contrast, the nuclear Maximum Clade Credibility (MCC) trees obtained from the post-burnin posterior tree distributions revealed strongly supported conflicting topologies across scaffolds. The most common topology among MCC trees, obtained from seven nuclear scaffolds with strong support, was the expected species topology *P. theophrasti* (*P. sylvestris* (*P. dactylifera*)) (fig. 5 and supplementary table S8, Supplementary Material online), reflecting previous findings (e.g., Gros-Balthazard et al. 2017; Flowers et al. 2019; Mohamoud et al. 2019, but see below). These analyses yielded an age of divergence of ∼3.2 Ma between North African and Asian populations of *P. dactylifera* and an age of 8 My for the MRCA of *P. sylvestris* and *P. dactylifera*, whereas the split of *P. theophrasti* from the MRCA of *P. sylvestris* and *P. dactylifera* was estimated to have occurred 10 Ma (fig. 5 and supplementary table S8, Supplementary Material online).

Analyses from the remaining 11 scaffolds yielded two alternative topologies: in the MCC trees from seven of these scaffolds, the relationship Asian *P. dactylifera* (*P. theophrasti* + North African *P. dactylifera*) uncovered by Flowers et al. (2019) was strongly supported, whereas the MCC trees from the remaining four scaffolds displayed a topology were *P. theophrasti* was closer to both North African *P. dactylifera* and Asian *P. dactylifera* than *P. sylvestris* (fig. 5*B*). The former analyses yielded an age of ∼3.4 My for the MRCA of *P. theophrasti* and North African *P. dactylifera*, whereas the split between *P. theophrasti* and both populations of *P. dactylifera* recovered in the latter was dated to be 5.8 My old.

Secondly, to quantify the frequencies of all three possible quartets formed by *P. reclinata*, *P. theophrasti*, *P. sylvestris*, and *P. dactylifera*, we subsampled scaffold alignments to include one individual per population (individuals SRR121596, SRR5120114, SRR8400845, SRR8400846, SRR8400855) and split them into blocks of 10,000 nonoverlapping bases. ML phylogenetic trees were estimated from each block, and we computed the relative frequencies of all possible alternative topologies across the genome and for each scaffold independently. We assumed that the true species tree is *P. theophrasti* (*P. sylvestris* (*P. dactylifera*)), which is corroborated by our ASTRAL and Bayesian MSC analyses (fig. 5*A* and supplementary fig. S5, Supplementary Material online), although Gros-Balthazard et al. (2021) only found this topology after exclusion of all North African date palms and an admixed Asian sample. With these samples included, the relationship instead was *P. sylvestris* (*P. theophrasti* (*P. dactylifera*)) (l.c., supplementary fig. S6, Supplementary Material online) as also found in our figure 5*B*. Regardless of the statistical support threshold at branches (see Materials and Methods), we found that the quartets congruent to the species tree topology were the most frequent genome-wide and across all but two of the scaffolds (supplementary fig. S6, Supplementary Material online). Moreover, the quartet supporting a closer relationship between *P. dactylifera* and *P. theophrasti* was recovered as the second most frequent in all scaffolds as compared with the third possible alternative quartet. Such distribution of frequencies for the tree quartets suggests a scenario where phylogenetic conflict is the product of gene flow between *P. theophrasti* and *P. dactylifera* rather than ILS. Likewise, the frequencies of all 15 possible topologies formed by *P. theophrasti*, *P. sylvestris*, and *P. dactylifera*, with *P. reclinata* as outgroup indicated that topologies supporting the species tree were by far the most frequent (58%), followed by those supporting a closer relationship of *P. theophrasti* and North African individuals of *P. dactylifera* (24%). The third most frequent topology was that supporting the sister relationship of *P. theophrasti* to *P. dactylifera* (8.13%; fig. 5*C* and *D*; supplementary table S9, Supplementary Material online). The remaining trees supporting eight alternative topologies attained a joint frequency of 9%, none of which occurred in high proportions (supplementary table S9, Supplementary Material online). Some of these topologies suggested a closer relationship between North African and Asian individuals of *P. datcylifera* to *P. sylvestris*.

Lastly, to statistically test for the presence of introgressed sequences among date palm populations, we compared the minimum pairwise distance between simulated and empirical data sets as implemented in the software JML v.1.3.1 (Joly 2012), using as a framework the posterior distribution species trees obtained by StarBEAST2 for each nuclear scaffold. The test revealed significant (Bonferroni adjusted *P *<* *0.05; supplementary table S10, Supplementary Material online) widespread gene flow between North African-Asian populations of *P. dactylifera, P. sylvestris* and *P. theophrasti*, thus providing support to the findings produced by our D-statistic analyses conducted to population level (supplementary tables S6 and S7, Supplementary Material online). Our multiple lines of evidence (i.e., D-statistics, tree quartet, and topology frequencies, JML) rules out ILS and indeed points toward gene flow as the main driver for the close relationship of *P. theophrasti* to the stem lineage of *P. dactylifera*.

## Discussion

Generating genomic data from plant archeological remains of known origin and unequivocal age provide a unique window into the timing and chronology of plant crop domestication and expansion processes (Gutaker and Burbano 2017; Swarts et al. 2017; Pont et al. 2019; Scott et al. 2019; Gros-Balthazard et al. 2021). The occurrence and timing of genetic exchanges between date palms and their wild relatives, *P. sylvestris* in Asia and *P. theophrasti* in Crete and Turkey, provide prime examples of the power of this approach. Though low in overall proportion, we retrieved sufficient genetic information from the endogenous aDNA of an ancient date palm leaf to test the timeline for introgression from two close relatives in the date palm genome.

Comparisons of our plastid and nuclear topologies and population structure analysis provide robust evidence for the genomic affiliation of the Saqqara leaf with modern North African *P. dactylifera* populations, as well as the occurrence of ancient gene flow between *P. dactylifera, P. sylvestris*, and *P. theophrasti.* The clustering of Asian *P. sylvestris* with selected North African date palm cultivars in plastid phylogenies has been previously reported (Pintaud et al. 2013; Chaluvadi et al. 2019; Flowers et al. 2019; Mohamoud et al. 2019), thus opening the question of whether gene flow or ancestral polymorphisms are responsible for this pattern (Flowers et al. 2019). The geographic ranges of these species today overlap only in northern India and Pakistan (fig. 2*C*). Notably, Gros-Balthazard et al. (2021), with a denser sampling of *P. dactylifera* and *P. theophrasti* than used here, found a species relationship of *P. sylvestris* (*P. theophrasti* (*P. dactylifera*)), when North African date palm samples were included, but *P. theophrasti* (*P. sylvestris* (*P. dactylifera*)) with North African samples excluded, pointing to perhaps even more complex gene flow patterns (cf. our fig. 5).

Extensive sequencing of over 200 organellar genomes of *P. dactylifera* has revealed that date palm cultivars contain four haplotypes that are tightly linked to the geographical origin of the cultivar (Mohamoud et al. 2019), but with largely unknown diversification times. In particular, one major haplotype (NA1, see *fig. 2* in Mohamoud et al. [2019]) that includes mostly cultivars of North African origin is highly divergent from the remaining haplotypes and is shared with *P. sylvestris* (Mohamoud et al. 2019). The trace of gene flow between the Saqqara leaf, modern individuals of *P. dactylifera*, and *P. sylvestris* detected by our introgression analyses suggests that the recurrent clustering patterns of individuals from both species in plastid phylogenies could be derived from one or several chloroplast-capture processes mediated by hybridization. By confidently placing the Saqqara leaf plastid genome in the NA1 haplotype, we set a minimum age for the origin of this plastid subpopulation to *c.* 2,100 years BP. In addition, we estimate the North African *P. dactylifera*/*P. sylvestris* clade to have originated as early as ∼2 Ma (supplementary fig. S4 and table S8, Supplementary Material online). The trace of gene flow in the Saqqara leaf genome from the Asian *P. sylvestris* by 2,100 years suggest either that humans brought oriental cultivars to Egypt that already contained *P. sylvestris* ancestry or, alternatively, that the *P. sylvestris* range in the past extended from the West Himalayas across the Arab Peninsula to Egypt. A larger population-genomic sampling might help resolving this question.

In the Nile valley, date stones have been recovered from at least seven archeological sites in Egypt and three in Sudan spanning the Middle Kingdom (2,500–1,650 B.C.E.) to second intermediate period, but they become only common from the New Kingdom (1,570–1,070 B.C.E.) onward (see reviews in Zohary et al. [2015], and database in Flowers et al. [2019]). The importance of date culture during the New Kingdom is also reflected artistically, for instance in garden scenes within tomb wall paintings (Parkinson 2008). More limited evidence from the Old Kingdom (2,700–2,100 B.C.E.) includes findings of two date stones, and occasional fragments of other plant parts, from Giza (Malleson and Miracle 2018). Textual evidence from the Old Kingdom additionally refers to imported dates (Tallet 2017). Some early finds may therefore represent either imports or cultivated trees (Gros-Balthazard and Flowers 2021). Examples of date stones recovered from earlier pre-Dynastic sites, notably from El Omari and Hierakonpolis, however, might be intrusive and lack reliable context (Friedman R, personal communication to. O.A.P.E., March 1, 2020) (Flowers et al. 2019). Lastly, potential date palm leaves and fiber, mostly in funeral contexts, are known from around 3,800 B.C.E. onward (Vartavan et al. 1997).

Further west of the Nile valley, evidence for date cultivation in Libya, at Zinkekra, comes from the early first millennium B.C.E. and is an early example of oasis agriculture in North Africa (Van der Veen and Westley 2010; Pelling 2013). Additionally, a date stone dating to c.1400–1300 B.C.E. from the Wadi Tanzzuft, some 400 km further south-west, suggests date cultivation in the central Sahara by that time (di Lernia and Manzi 2002; Mattingly and Wilson 2010).

Archeobotanical and historical records (such as the ones above) provide minimum ages for relevant events (Ramos-Madrigal et al. 2016; Swarts et al. 2017; Gutaker et al. 2019; Scott et al. 2019; Gros-Balthazard et al. 2021), whereas molecular clock-dated phylogenomic frameworks can provide the divergence times of crops from close or distant living relatives (Pease and Hahn 2015). Our study contributes for the first time a densely sampled time tree for the genus *Phoenix*, which reveals that gene flow from *P. theophrasti* to *P. dactylifera* might have occurred already when the species diverged during the late Miocene, prior to the divergence of North African and Asian populations of *P. dactylifera* and its domestication. This maximum timestamp for the gene flow of *P. theophrasti* and *P. dactylifera* coincides with the establishment of the first aridification period of North Africa (Pickford et al. 2006; *fig. 1* in Zhang et al. [2014]), a phenomenon that drove the expansion of semiarid and arid climates that ultimately promoted drastic changes in the distribution of floras in the region (Douady et al. 2003; Feakins et al. 2013).

Modern hybrid zones between *P. dactylifera* and *P. sylvestris* are known, and the species also hybridize in botanical gardens and plantations (Gros-Balthazard 2013). The small but consistent proportion of alleles shared between the Saqqara date palm and *P. theophrasti*, and *P. sylvestris* (fig. 4) provides evidence that hybridization between these three species had already occurred ∼2,100 years BP. Nevertheless, caution is required when interpreting our D-statistics because the number of homologous bases analyzed (ranging from hundreds to thousands; supplementary tables S4–S7, Supplementary Material online) represents only a small fraction of the nuclear genome. A better representation of the Saqqara nuclear genome, ideally attained through target capture to increase the proportion of endogenous nuclear DNA, would help to further define the proportion of this genome that has been inherited from *P. theophrasti* or any other wild relative. Alternatively, the early implementation of clonal propagation of offshoots derived from hybrid individuals could have contributed to the survival of admixed genotypes. Archeological evidence supporting such agricultural practice, however, is lacking.

## Conclusion

Through a combination of molecular clock approaches and successful retrieval of ancient DNA from archaeobotanical objects of date palm, this study provides maximum and minimum timestamps for the occurrence of introgression processes in the evolutionary history of date palm. Our plastid and nuclear topological frameworks, together with the genomic composition analysis involving different reference genomes, levels of contiguity and genomic representations, consistently indicate that the Saqqara palm belongs to the same clade as the modern North African *P. dactylifera*. The ancient introgression from two wild relatives into domesticated date palm also prompts the investigation of the functional significance of introgressed *P. theophrasti* and *P. sylvestris* genes, which in turn could prove useful in date palm breeding.

## Materials and Methods

### Plant Taxon Sampling

Our sampling builds upon the population genomic studies of Flowers et al. (2019) and Gros-Balthazard et al. (2017). We sampled 17 individuals of wild and cultivated Asian and North African *P. dactylifera* populations, as well as 18 accessions of five closely related species, namely *P. atlantica*, *P. canariensis, P. reclinata, P. sylvestris*, and *P. theophrasti* (supplementary table S2, Supplementary Material online), following the accepted taxonomy (Barrow 1998; Gros-Balthazard, Baker, et al. 2021). In addition, a shallow genomic representation of the New Guinean palm *Licuala montana* was sequenced to produce a plastid genome assembly that was subsequently used as the outgroup for phylogenetic analyses. This species was chosen due to the sister relationship between the palm tribes Phoeniceae (containing *Phoenix*) and Trachycarpeae (containing *Licuala*) (Baker and Dransfield 2016).

Whole-genome sequence data from these taxa were obtained from the NCBI Sequence Read Archive repository. Twenty million reads were downloaded for each accession using the tool *fastq-dump* of the SRA toolkit. Nearly all accessions sampled are linked to vouchers and have known origins (Gros-Balthazard et al. 2017; Flowers et al. 2019); detailed information on their provenance, average read length and number of bases downloaded are provided in supplementary tables S1 and S2, Supplementary Material online.

### Radiocarbon Dating and Ancient DNA Extraction

To determine with accuracy the age of the Saqqara date palm item (accession EBC 26796), 1 cm^2^ of leaf removed from the edge of the sample was sent to the Laboratory of Ion Beam Physics, ETH-Zurich. The leaf sample underwent a treatment with solvents and acid–base–acid washes (Hajdas 2008) to remove potential contamination of waxes, carbonates, and humic acids. The dry, clean material (weighing 2.6 mg, equivalent to 1 mg of carbon) was weighed into tin cups for combustion in the Elemental Analyzer for subsequent graphitization (Němec et al. 2010). The resulting graphite was pressed into aluminium cathodes and the ^14^C/^12^C and ^13^C/^12^C ratios were measured using the Mini Carbon Dating System dedicated accelerator mass spectrometry facility (Synal et al. 2007). The radiocarbon age was calculated following the method described by Stuiver and Polach (1977) using the measured ^14^C content after correction for standards, blank values, and fractionation (*δ*^13^C values were measured semisimultaneously on graphite). The reported conventional age in years BP (before 1950 AD or CE) was calibrated to a calendar age using OxCal version 4.2.4 (Reimer et al. 2013) and the IntCal13 atmospheric curve (Ramsey and Lee 2013).

Ancient DNA (aDNA) was extracted by grinding a small piece of a leaflet (<1 cm^2^, 5.8 mg) with a Retsch mill (MM 400). DNA extraction was performed following the modified protocol of Wales et al. (2014) (Dabney et al. 2013; Pedersen et al. 2014). For the digestion treatment, a lysation buffer containing 0.5% (w/v) N-lauroylsarcosine (Sigma Aldrich L9150-50G), 50 mM Tris–HCl (Thermo Fisher Scientific 15568025), 20 mM EDTA (VWR E177-500MLDB) 150 mM NaCl (Thermo Fisher Scientific AM9760G), 3.3% 2-mercaptoethanol (Sigma Aldrich 63689-25ML-F), 50 mM DL-dithiothreitol (Sigma Aldrich D9779-250MG), and 0.25 mg/ml Proteinase K (Promega V3021) was applied to the leaflet powder as described in Wales et al. (2014). DNA purification was performed according to Dabney et al. (2013) but with reduced centrifugation speed (450× g), following Basler et al. (2017).

### Ancient DNA Library Preparation and Sequencing

A genomic Illumina library was prepared from the extracted aDNA following the single-stranded protocol of Korlević et al. (2015). The protocol included a treatment with Uracil-DNA-Glycolase (New England Biolabs M0279) to remove uracil residues and Endonuclease VIII (New England Biolabs M0299) to cleave DNA strands at abasic sites. Circligase II (2.5 U/µl; Biozym 131406) was used for the fill-in reaction which was carried out overnight. A quantitative PCR was performed on a PikoReal 96 Real-Time PCR machine (Thermo Fisher Scientific TCR0096) using 0.2% of the unamplified library and the following thermal profile: 10 min initial denaturation step at 95 °C, followed by 40 cycles of: 15 s at 95 °C, 30 s at 60 °C, and 1 min at 72 °C. The quantitative PCR reaction mix contained a final volume of 10 µl:1 µl of diluted library, 1× SYBR Green qPCR Master Mix (Applied Biosystems 4309155), 0.5 µM of each primer IS7 and IS8. Three replicates of each library were used. Indexing PCR was performed by the appropriate number of cycles according to the results of the qPCR, with 8 bp indices added to the 5′ and 3′ adapters. The PCR and final concentrations used were the same as described by Gansauge and Meyer (Korlević et al. 2017), but with a final volume of 80 µl using 20 µl of template. DNA sequencing was performed on an Illumina NextSeq 500 sequencing platform, using the 500/550 High Output v2 kit (75 cycles, Illumina FC-404-2005), with a custom read-1 (Pedersen et al. 2014) and a custom index-2 (Paijmans et al. 2017) sequencing primers. All extractions and library preparations were performed in the ancient DNA facility of the University in Potsdam; negative controls were included in all steps. The newly generated sequence read data of the Saqqara leaf genome have been made available in the Sequence Read Archive project PRJNA739191.

### Genome Skimming of *L. montana*

We extracted genomic DNA from silica-dried leaf tissue of *L. montana* using the Qiagen DNeasy Plant kit, following the manufacturer’s protocol. A genomic Illumina paired-end library was prepared using the NEBNext Ultra II library preparation kit, following the manufacture’s protocol and with an average insert size of 464 bp. Library sequencing was performed by the company Genewiz (NJ) on a HiSeq platform. A total of four million paired-end reads were produced. The newly generated sequence read data of *L. montana* have been made available in the Sequence Read Archive project PRJNA739191.

### High-Throughput Read Data Processing

The Illumina raw reads were quality filtered using Trim Galore v.0.4 (Krueger 2015), discarding sequences with an average phred33 score below 20 and a length <25. Pre- and posttrimming read quality was assessed using FASTQC v.0.1 (Andrews et al. 2015). The proportion of endogenous DNA sequences present in the ancient Saqqara date palm leaf extract was assessed by mapping the trimmed read data against two nuclear genomes (Khalas variety: assembly GCA000413155.1, Al-Mssallem et al. 2013; Bahree cultivar: assembly GCA0009389715.1, Hazzouri et al. 2019) and one plastid genome of *P. dactylifera* (assembly NC013991; Yang et al. 2010) using Magic-BLAST v.1.5.0 (Boratyn et al. 2019), a word size value of 18 (*-word_size*), and a penalty for nucleotide mismatch (*-penalty*) and score (*-score*) threshold of 4 and 20, respectively. The full reports of our Magic-BLAST analyses are freely available in supplementary file S1, Supplementary Material online. We then determined the proportion of nuclear/plastid ancient date palm read data sequenced by filtering the number of hits mapped by Magic-BLAST onto nuclear and organellar scaffolds.

Given the low proportion of nuclear genomic data recovered from the ancient Saqqara date palm leaf (see Results), we mapped trimmed reads of both modern and ancient accessions on nuclear and plastid targeted scaffolds (i.e., scaffolds with Saqqara date palm leaf reads mapped). These represented 198 contigs, or 26.8% (149.01 Mb) of the *P. dactylifera*’ nuclear genome assembly. We investigated the proportion and position of mis-incorporated nucleotides in the Saqqara date palm leaf DNA following the protocol of Latorre et al. (2020), that is, using aligned aDNA reads and the tool mapDamage2 v.2.0.9 (Jónsson et al. 2013). We compared nucleotide mis-incorporation patterns between the aligned aDNA reads of the Saqqara date palm leaf and DNA reads of modern date palm accessions (SRR5120110). Finally, to reduce the fraction of mis-incorporated nucleotides mapped onto the reference genome, which were found to occur with higher frequency on the first 1–3 positions of DNA fragments (fig. 1 and supplementary fig. S1, Supplementary Material online), we trimmed two bases at the 3′ and 5′ end of the aDNA reads, using Trim Galore v.0.4. Read mapping, alignment, and DNA damage analyses were implemented through the pipeline PALEOMIX v.1.2.13 (Schubert et al. 2014). The trimmed read data were mapped using the software bowtie v.2.3.4.1 and a mapping quality threshold of 20, followed by a realigning step around indels and filtering of duplicated reads with the software GATK v.3.8.1 (McKenna et al. 2010) and Picard-tools v.1.137 (Thomer et al. 2016). Read mapping, and average coverage statistics for each accession sampled in this study are provided in supplementary table S1, Supplementary Material online.

Mis-incorporated nucleotides in DNA fragments are characteristic of sequence data derived from historical and archaeobotanical specimens (Estrada et al. 2018). To account for biases in the mapping of aDNA read data onto the reference genome (Günther and Nettelblad 2019) and test the robustness of our population genomic inferences against missing data (Skotte et al. 2013), we also mapped the ancient and modern DNA reads onto 18 highly contiguous scaffolds of a newly assembled nuclear genome of *P. dactylifera* (four-generations backcross of a Bahree cultivar, assembly GCA0009389715.1; Hazzouri et al. 2019), representing 50% of the nuclear genome (∼380 Mb). Read mapping and alignment were conducted using the same procedure and tools as specified above.

Additionally, to determine whether mis-incorporated nucleotides could potentially affect downstream analyses involving the Saqqara date palm leaf, we compared the transition/transversion ratios (Ti/Tv) and error rates (i.e., excess of derived alleles compared with the number of derived alleles of a modern accession; Kim et al. 2011) of our filtered 2 bp trimmed ancient DNA sample with that of a panel of 24 modern samples. If mis-incorporations are pervasive in the aDNA fragments, a deviation of Ti/Tv and error rates is expected when compared with values derived from modern accessions. Ti/Tv ratios were computed by first obtaining a matrix of 7,599 called genotypes from genotype likelihoods (GLs) using the software PLINK v.1.9 (Purcell et al. 2007). The GLs were obtained from read mappings against the *P. dactylifera* Bahree cultivar reference genome and retaining only sites that were common across all sets of modern individuals and the Saqqara date leaf (see Population Structure, Nuclear Phylogenomics, and Gene Flow of the Saqqara Date Palm). The resulting 7,599 genotyped sites were then used as input in vcftools v.0.1.16 (Danecek et al. 2011) to produce the Ti/Tv ratios. Error rates were computed for the same set of samples in ANGSD v.0.929 (Korneliussen et al. 2014), using the function *doAncError* (option -1) and, as input, corresponding reads mapped against the *P. dactylifera* Bahree cultivar reference genome together with the reference of a modern individual known to be free of nucleotide mis-incorporations (SRR5120110; see supplementary fig. S1, Supplementary Material online). Such reference was computed in ANGSD using the function *doFasta* (option -1) by sampling a random base at each position following the estimation of allele frequencies, a minimum base quality score of 25 (-*minQ*) and minimum sequencing depth of 5 (-*setMinDepth*).

### Plastid Phylogenomic Analyzes of *Phoenix*

Plastid genomes in angiosperms are usually uniparentally inherited (Hageman 2004; Wicke et al. 2011) and unlike the nuclear genome, their replication does not involve recombination (Reboud and Zeyl 1994). However, reports of multiallelic positions in plastid genomes suggest that heteroplasmy might be a frequent phenomenon (Sabir et al. 2014). To account for the potential occurrence of multiallelic positions, we produced consensus plastid genome sequences of modern date palm accessions from the BAM files produced by PALEOMIX by following the modified statistical base-calling approach of Li et al. (2008), that is, minimum read depth of 10, and bases matching at least 50% of the reads overlapping a particular position, with “Ns” being called for sites that do not fulfil these conditions (Yang et al. 2010). Because the attained average coverage of the Saqqara date palm leaf plastid genome was ∼2× (supplementary table S1, Supplementary Material online), the consensus plastid sequence for this accession was produced by using a minimum read depth of 2, bases matching at least 50% of the reads overlapping a given position and missing data represented as Ns whenever parts of the reference plastid genome were not covered by aDNA reads. The whole-plastid genome consensus sequences were produced in Geneious v.8.0. Consensus plastid genome sequences were aligned with Mauve using a progressive algorithm and assuming collinearity (Darling et al. 2004). The resulting ∼150,000-bp alignment was first trimmed to exclude mis-aligned regions and positions with >90% missing data (final alignment length of 103,807 bp) and then subjected to ML tree inference in RAxML v8.0 (Stamatakis 2014), using the GTR substitution model, 25 gamma rate categories, and 1,000 bootstrap replicates. The number of informative sites and proportion of missing data of the whole-plastid genome alignment are available in supplementary table S3, Supplementary Material online.

### Population Structure, Nuclear Phylogenomics, and Gene Flow of the Saqqara Date Palm

Given the low-depth of the high-throughput sequencing data produced for the ancient Saqqara date palm leaf, we relied on GL to place the aDNA nuclear genomic data of the Saqqara date in range with genomic sequences of modern *Phoenix* samples. We computed nuclear GL using the software ANGSD v.0.929 (Korneliussen et al. 2014), by implementing the GATK GL model, inferring the minor and major alleles, and retaining polymorphic sites with a minimum *P* value of 1-e^6^. To place the Saqqara date among modern samples, we first produced pseudohaploidized consensus sequences of 18 scaffolds for 24 samples representing *P. reclinata, P. sylvestris*, *P. theophrasti*, and Asian and North African populations of *P. dactylifera*. We used as input the reads mapped against the *P. dactylifera* Barhee reference genome (see High-Throughput Read Data Processing). The pseudohaplotypes were obtained in ANGSD, using the function doFasta by randomly sampling one base per position (option -*1*) following the estimation of the most common base while applying a minimum base quality score of 25 (-*minQ*) and minimum sequencing depth of 2 (-*setMinDepth*). Whenever a position did not fulfil any of the requirements specified above, an ambiguity (N) was called, thus producing pseudohaplotypes of equal length for each individual. Such approach has been widely used in studies involving the analysis of modern and ancient DNA samples in phylogenomic frameworks (see Sheng et al. 2019; Westbury et al. 2020). The resulting alignments were then concatenated into a supermatrix and subsequently trimmed in Geneious v. R.8.0 to consider only sites that were homologous to those retrieved from the Saqqara nuclear genome. We computed a NeighborNet network derived from uncorrected P-distances using the software SplitsTree4 (Huson 1998) and the supermatrix as input. The support at edges was estimated by conducting 1,000 bootstrap replicates (supplementary file S3, Supplementary Material online). The number of informative positions and proportions of missing data for this alignment are provided in supplementary table S3, Supplementary Material online.

We next conducted principal component (PCA) and population structure analyses using nuclear GLs and the tools PCangsd (Meisner and Albrechtsen 2018), and NGSadmix (Skotte et al. 2013) of the software ANGSD, respectively. Because PCA can be particularly affected by the proportion of overlapping sites between modern and ancient populations (Ausmees 2019), we computed covariance matrices by using only GLs derived from sites that were shared across all the modern individuals and the ancient Saqqara date palm leave (i.e., option -*minInd* set to 35), and a maximum of 1,000 iterations. Admixture analyses were conducted with number of population (K) set from two to eight and a maximum of 20,000 iterations. The best K was selected by comparing the resulting likelihood values derived from each K iteration.

To test for admixture between the Saqqara date palm and other lineages amongst date palms or closely related species (*P. atlantica*, *P. sylvestris*, *P. theophrasti*), we used the D-statistic framework as implemented in the software ANGSD. To account for the differences in sequencing coverages obtained for modern individuals and the Saqqara leaf and to assess the robustness of our introgression tests, two different approaches were followed, namely: 1) between nuclear genomes of each individual (i.e., sampling one base from reads of one individual per population; Korneliussen et al. 2014) and 2) between populations (i.e., considering all reads from multiple individuals in each population; Soraggi et al. 2018). In the latter analyses, we followed the same sampling strategy of Flowers et al. (2019), thus excluding two individuals of *P. sylvestris* and one of *P. theophrasti* that were reported as potentially interspecific hybrids that could potentially bias the count of ABBA/BABA sites within a population. Nuclear GL were used as input and D-statistics were computed for both tests. In (1), D-statistics were calculated by sampling a random base at each analyzed position in blocks of five million base pairs, removing all transitions to rule out possible postmortem base misincorporations together with reads with qualities lower than 30, and setting *P. reclinata* as a fixed outgroup terminal following the same experimental design as in Flowers et al. (2019). In (2), individuals were assigned to six populations defined according to their species identity, for example, all modern individuals of *P. dactylifera* were assigned to one population (supplementary table S2, Supplementary Material online); the Saqqara leaf was assigned to its own population as well as the outgroup (*P. reclinata*), following recommendations of Soraggi et al. (2018). D-statistics were then calculated by sampling reads from multiple individuals in each population. Moreover, to account for the influence of polymorphisms in the outgroup taxon, we executed population tests using polymorphic and nonpolymorphic sites (supplementary table S6, Supplementary Material online) and only nonpolymorphic sites in the outgroup (“–enhance” flag in ANGSD; supplementary table S7, Supplementary Material online). The analysis considering only nonpolymorphic sites yielded nearly identical results (supplementary table S7, Supplementary Material online) to those obtained whenever all sites where included (supplementary table S6, Supplementary Material online) albeit with reduced statistical significance due to the limited number of sites analyzed whenever the Saqqara sample was involved. As such, discussions regarding gene flow between the Saqqara leaf and modern populations of *Phoenix* are based only on the test considering all sites. The significance of the analyses was assessed by executing a block-Jacknife test which derived SEs and *Z* scores, using a block size of 5 Mb. For population level analyses, *P* values were derived. The admixture of the Saqqara date palm was discussed based solely on significant *P*- and D-statistic values (i.e., |*Z*| > 3). D-statistic values, SDs, *Z* scores, and the number of evaluated sites for each topological permutation are provided in supplementary tables S4–S7, Supplementary Material online. NGSadmix and D-statistic analyses were conducted on filtered GLs derived from read data mapped on: 1) 198 contigs representing 26.8% of the *P. dactylifera* genome (assembly GCA000413155.1); and 2) 18 scaffolds representing 50% of the nuclear genome of *P. dactylifera* (assembly GCA0009389715.1, see High-Throughput Read Data Processing section of Materials and Methods above).

### Genome-Wide Nuclear Phylogenomics and Absolute Age Estimations of Divergences in *Phoenix*

To better characterize the absolute timing of gene and lineage divergence in *Phoenix*, we produced a genome-wide phylogeny of a subset of our modern samples using ML and Bayesian methods. To identify possible events of reticulation in the genus, we produced pseudohaploidized consensus sequences of the 18 nuclear scaffolds for 12 samples representing *P. reclinata* (sample SRR8400855)*, P. sylvestris* (SRR5120114, SRR6439420, SRR8400843), *P. theophrasti* (SRR8400849, SRR8400846, SRR8400847), and Asian (SRR5120112, SRR121596, SRR121612) and North African (SRR8400852, SRR5120109) populations of *P. dactylifera* (i.e., defined by the outcome of admixture analyses, see Results) following the same approach discussed earlier with the exception of setting the option -*setMinDepth* to 5 (see Population Structure, Nuclear Phylogenomics, and Gene Flow of the Saqqara Date Palm). A total of 18 alignments were produced (supplementary file S4, Supplementary Material online).

To reduce the influence of missing data on phylogenetic analyses, we trimmed positions including more than 10% of gaps in each alignment, which subsequently were split into nonoverlapping blocks of 10,000 bp. This approach was chosen to account for potential differences in the evolutionary histories of loci along each scaffold which ultimately can lead to producing contrasting tree topologies, while allowing for a number of loci sufficiently low to render computational analyses tractable. The total number of blocks and their corresponding features, including number of informative positions and proportions of missing data, are provided in supplementary table S3, Supplementary Material online. ML phylogenies were inferred from each block, using the same settings mentioned above to infer the plastid genome phylogeny (see Plastid Phylogenomic Analyses of *Phoenix*). We employed the resulting 1,143 ML phylogenies to infer a species tree under an MSC framework as implemented in the software ASTRAL-III v.5.6 (Mirarab and Warnow 2015), after collapsing branches with less than 10% of LBS, as recommended in Zhang et al. (2018).

To determine the frequency of gene tree quartets supporting (LBS = 0 and >90) the species tree or any other alternative relationship, we compared each ML tree produced from 10,000 bases blocks against the species tree using the software DiscoVista v.1.0 (Sayyari et al. 2018). Each internal branch of a resolved, bifurcating tree has four taxa (or groups of taxa) around itself, and there are only three possible topologies for the arrangement of these four taxa around the branch; these three topologies are often referred to as three alternative quartets. For each internal branch in the species tree, DiscoVista computes the relative frequencies at which the three alternative quartets are found in the gene trees, thereby providing a measure of the relative support for each alternative. The analysis was performed by first looking at conflict among all 1,143 blocks, and then separately for each of the 18 scaffolds to test for conflict among the blocks derived from each scaffold.

Inferring absolute ages of divergence in the presence of gene tree discordance is challenging because distinct evolutionary histories can lead to differences in estimated ages of divergence (Widhelm et al. 2019). We relied on the program StarBEAST2 v.2.5 (Ogilvie et al. 2017) to estimate absolute ages of lineage divergence from nuclear and plastid genomic data, using the same taxon sampling employed to calculate ML trees across the 18 scaffolds. The software implements MSC methods in conjunction with various molecular clock models, allowing species tree calibration in the presence of reticulation. One downside of the analysis is that it becomes time intractable whenever large numbers of partitions are being analyzed (Ogilvie et al. 2017; Widhelm et al. 2019; Mello et al. 2021). To render the Bayesian time estimations tractable, we independently analyzed one plastid and 18 scaffolds, the latter divided each in nonoverlapping blocks of 100,000 bases, with an average of 913 parsimony informative sites. The number of variable and parsimony informative sites, proportion of missing data and A/T, G/C content for nuclear and plastid alignments were obtained using the program AMAS v.1.0 (Borowiec 2016). The total number of plastid and nuclear blocks and their corresponding features are provided in supplementary table S3, Supplementary Material online.

To calibrate the plastid phylogeny of *Phoenix*, we applied a secondary calibration to the tree root marking the divergence between the Trachycarpeae (represented by the outgroup *L. montana*) and Phoeniceae tribes. The prior probability distribution of this constraint was specified using a Normal distribution with a mean value of 76 Ma and a SD of 1, reflecting the posterior age distribution obtained by Cano et al. (2018). Substitution rates were modeled with a GTR substitution model and rate heterogeneity among sites following a Gamma distribution with four categories and a relaxed log-normal molecular clock, with a uniform prior distribution for the mean rate, ranging from 1.0e-5 to 0.001 substitutions/site/Ma. The posterior estimates of the coefficient of variation (CV) of the substitution rate for the plastid and nuclear data sets indicated that using a lognormal relaxed molecular clock fit the data better than a strict clock (i.e., all CV >0.1; Drummond and Bouckaert 2015). The relaxed log-normal clock values were chosen to encompass previously estimated palm plastid substitution rates (Gaut et al. 1992). The ploidy level was set to 1 (option “Y or mitochondrial”) as recommended for organellar data sets (Drummond and Bouckaert 2015). These priors were applied in conjunction with Coalescent Constant Population tree model with a mean population size of 1.0 and a noninformative prior of 1/X, following Drummond and Bouckaert (2015) for situations when the taxon sampling includes a mix of several individuals per population. We ran StarBEAST2 for 100 million generations and sampled every 5,000 states, ensuring that all parameters reached convergence and large effective sample sizes (ESS >200; Ogilvie et al. 2017).

Given that nuclear-wide genome data are not available for *L. montana*, we relied on the ages of divergence of *Phoenix* derived from our plastid chronogram to calibrate the root of our nuclear tree (including *P. reclinata*, *P. sylvestris*, *P. theophrasti*, and individuals representing Asian and African populations of *P. dactylifera*; see Results). The prior probability distribution of the root age was set to follow a Normal distribution of arithmetic mean 12.5 Ma and SD of 1, reflecting the posterior age distribution yielded by our plastid analysis for this node. We used the same priors and models employed to obtain absolute ages from the plastid data set, with the exception that: 1) the mean rate of the relaxed molecular clock was set to a uniform distribution with lower and upper values of 0.0001 and 0.01, respectively, following Gaut et al. (1992) and Gros-Balthazard et al. (2017) while allowing substantial deviation from them; and 2) the ploidy level was set to 2 (option “autosomal nuclear”) as recommended for nuclear diploid data sets (Jatt et al. 2019). Nineteen ultrametric Maximum Clade Credibility (MCC) consensus trees were produced in TreeAnnotator v.2.5 (available at https://www.beast2.org/treeannotator/) for plastid and nuclear scaffolds based on the posterior tree distribution from each analysis using the median node heights option and a burn-in fraction of 10%. Median node ages and 95% highest posterior density intervals are provided in supplementary table S8, Supplementary Material online.

### Validation Tests of Gene-Flow Processes

To determine whether the conflicting, strongly supported relationships observed in our nuclear genome-wide ML and Bayesian phylogenies are the product of gene flow and not ILS, we conducted statistical tests involving the comparison of minimum pairwise distance between simulated and empirical data sets in the software JMLv.1.3.1 (Joly 2009). The program relies on posterior predictive checking to assess whether the minimum distance between any two given DNA sequences is smaller than expected assuming the absence of gene flow (Joly 2012). To this end, we employed 396,000 post-burnin randomly subsampled posterior trees from the dating analyses conducted on the 18 nuclear scaffold data sets (i.e., 22,000 trees per analysis) and their corresponding DNA alignments. A total of 396,000 simulations were run using an average mutation rate of 0.005 (option *locusrate*), a heredity scalar value of 1 as recommended for nuclear data sets (option *heredityscalar*), the GTR substitution model (option *seqgecommand*), and a *P* value of 0.05 (option *significancelevel*). The resulting *P* values were adjusted by implementing a Bonferroni correction. The minimum distances between the ingroup taxa and their statistical significance are provided in supplementary table S10, Supplementary Material online.

## Supplementary Material

Supplementary data are available at *Molecular Biology and Evolution* online.

## Supplementary Material

msab188_Supplementary_Data
