# Supplementary material for: Molecular Clocks and Archeogenomics of a Late Period Egyptian Date Palm Leaf Reveal Introgression from Wild Relatives and Add Timestamps on the Domestication
Source: Mol Biol Evol. 2021 Jun 30;38(10):4475–92. doi: 10.1093/molbev/msab188 (PMC8476131; doi:10.1093/molbev/msab188)
Supplement: msab188_Supplementary_Data [file msab188_supplementary_data.pdf]

Molecular Biology and Evolution - **Supporting Information**

Article title: Molecular clocks and archaeogenomics of a Late Period Egyptian date palm leaf reveal introgression from wild relatives and add timestamps on the domestication

Oscar A. Pérez-Escobar[1*], Sidonie Bellot[1*], Natalia A. S. Przelomska[1,2], Jonathan M. Flowers[3], Mark Nesbitt[1], Philippa Ryan[1], Rafal M. Gutaker[1], Muriel Gros-Balthazard[4], Tom Wells[5], Benedikt G. Kuhnhäuser[5], Rowan Schley[1], Diego Bogarín[6], Steven Dodsworth[1,7], Rudy Diaz[1], Manuela Lehmann[8], Peter Petoe[9], Wolf L. Eiserhardt[1,9], Michaela Preick[10], Michael Hofreiter[10], Irka Hajdas[11], Michael Purugganan[3], Alexandre Antonelli[1,12], Barbara Gravendeel[13], Ilia J. Leitch[1], Maria Fernanda Torres Jimenez[12], Alexander S.T. Papadopulos[14], Guillaume Chomicki[15‡], Susanne S. Renner[16‡], William J. Baker[1*‡]

Affiliations:
[1]Royal Botanic Gardens, Kew, Richmond TW9 3AE. London, UK.
[2]National Museum of Natural History, Smithsonian Institution, Washington, DC, USA.
[3]Center for Genomics and Systems Biology, New York University Abu Dhabi, United Arab Emirates.
[4] French National Research Institute for Sustainable Development, Montpellier, BP 64501 – 34394 Cedex 5, France.
[5]Department of Plant Sciences, University of Oxford, Oxford, OX1 3QU, UK.
[6]Lankester Botanical Garden, University of Costa Rica, San José, 302-7050, Costa Rica.
[7]School of Biological Sciences, University of Portsmouth, Portsmouth PO1 2DY, UK.
[8]British Museum, London, WC1B 3DG, UK.
[9]Department of Biology, Aarhus University, 8000 Aarhus C, Denmark.
[10]Institute of Biochemistry and Biology, University of Potsdam, 14469 Potsdam, Germany.
[11]Department of Earth Sciences, ETH Zurich, 8092, Switzerland.
[12]Gothenburg Global Biodiversity Centre and Department of Biological and Environmental Sciences, University of Gothenburg, 413 19, Sweden.
[13]Naturalis Biodiversity Center, Leiden, 2333, The Netherlands.
[14]Molecular Ecology and Fisheries Genetics Laboratory, School of Biological Sciences, University of Bangor, Bangor LL57 2UW, UK.
[15]Department of Animal and Plant Sciences, Alfred Denny Building, University of Sheffield, Western Bank, Sheffield S10 2TN, UK.
[16]Department of Biology, Washington University, Saint Louis, MO 63130, USA.

***Correspondence**: o.perez-escobar@kew.org (OAPE), s.bellot@kew.org (SB), or w.baker@kew.org (WJB).
‡Joint senior authors

The following Supporting Information is available for this article:

Supplementary Files S1-S4
Supplementary Figures S1-S6
Supplementary Tables S1-S10

File S1: Output of Magicblast analyses, revealing the proportion of nuclear and organellar endogenous ancient DNA content of the Saqqara leaf, as implemented using three reference genomes. The file is available at https://doi.org/10.6084/m9.figshare.14542125.

File S2: Maximum Likelihood analysis of whole plastome sequences showing the placement of the Saqqara specimen amongst *Phoenix* species. The file is available at https://doi.org/10.6084/m9.figshare.14542125.

File S3: Uncorrected P-distance split network with corresponding bootstrap support values produced from nuclear positions shared by the Saqqara specimen and modern accessions. The file is available at https://doi.org/10.6084/m9.figshare.14542125.

File S4: Plastid and nuclear alignments employed to estimate absolute times of divergence. The file is available at https://doi.org/10.6084/m9.figshare.14542125.

Supplementary Figures

Figure S1. Authentication of *Phoenix dactylifera* aDNA. DNA misincorporations for each nucleotide in a modern date palm genome (A) compared to the ancient Saqqara date palm leaf (B). X axes indicate individual nucleotide positions of DNA fragments. (Inset) read distributions lengths in a modern date palm genome (A) and the ancient Saqqara date palm leaf (B). (C) Error rates of the ancient Saqqara date palm leaf compared with those computed from modern accessions of *Phoenix*.

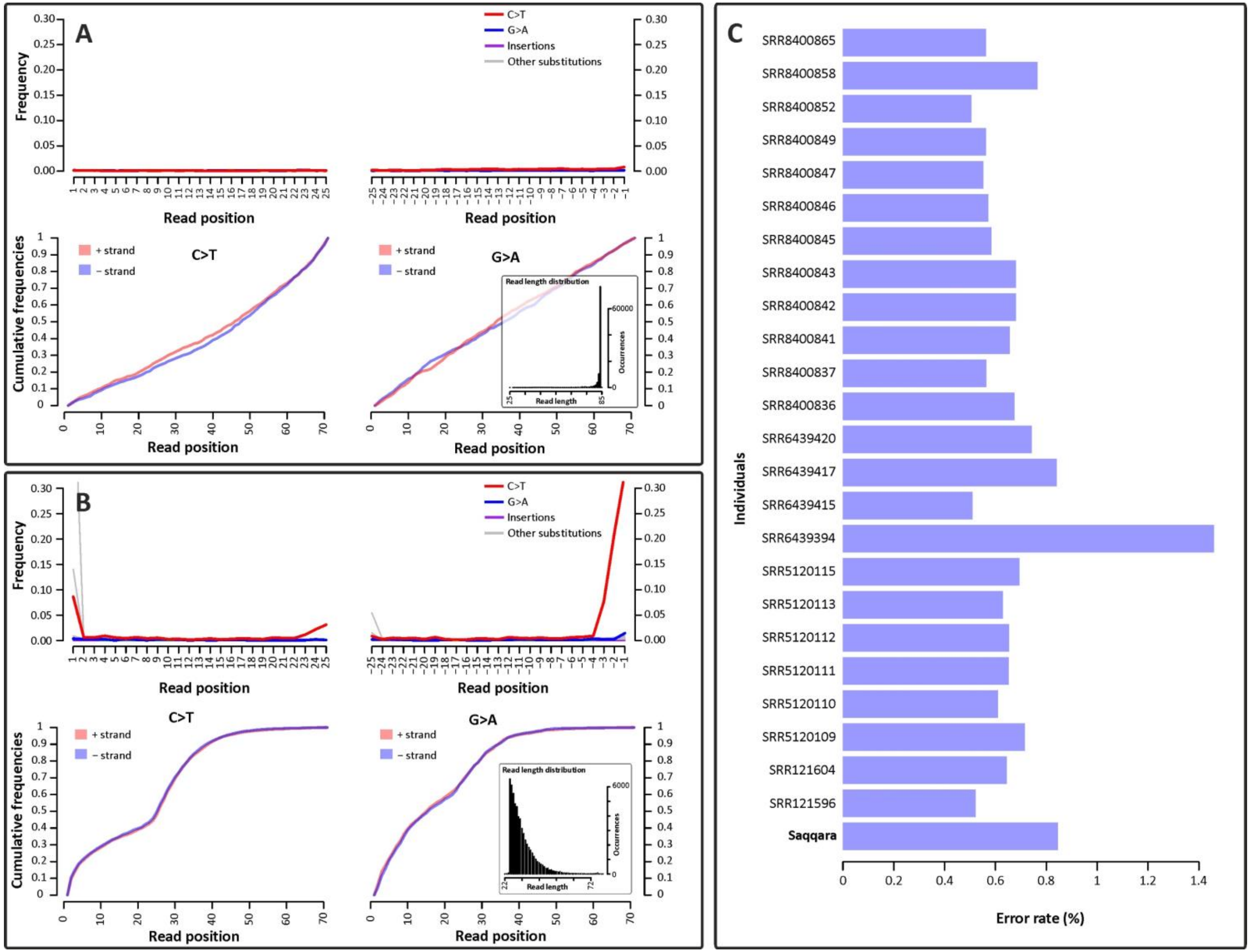

Figure S2. Cluster likelihood values derived from K 1-8 of population structure analyses based on estimated nuclear Genotype Likelihoods (GLs) derived from a highly fragmented (**[A]**, GCA000413155.1) and a highly contiguous reference genome (**[B]**, GCA0009389715.1). (Inset: delta log likelihood plots of cluster likelihood values).

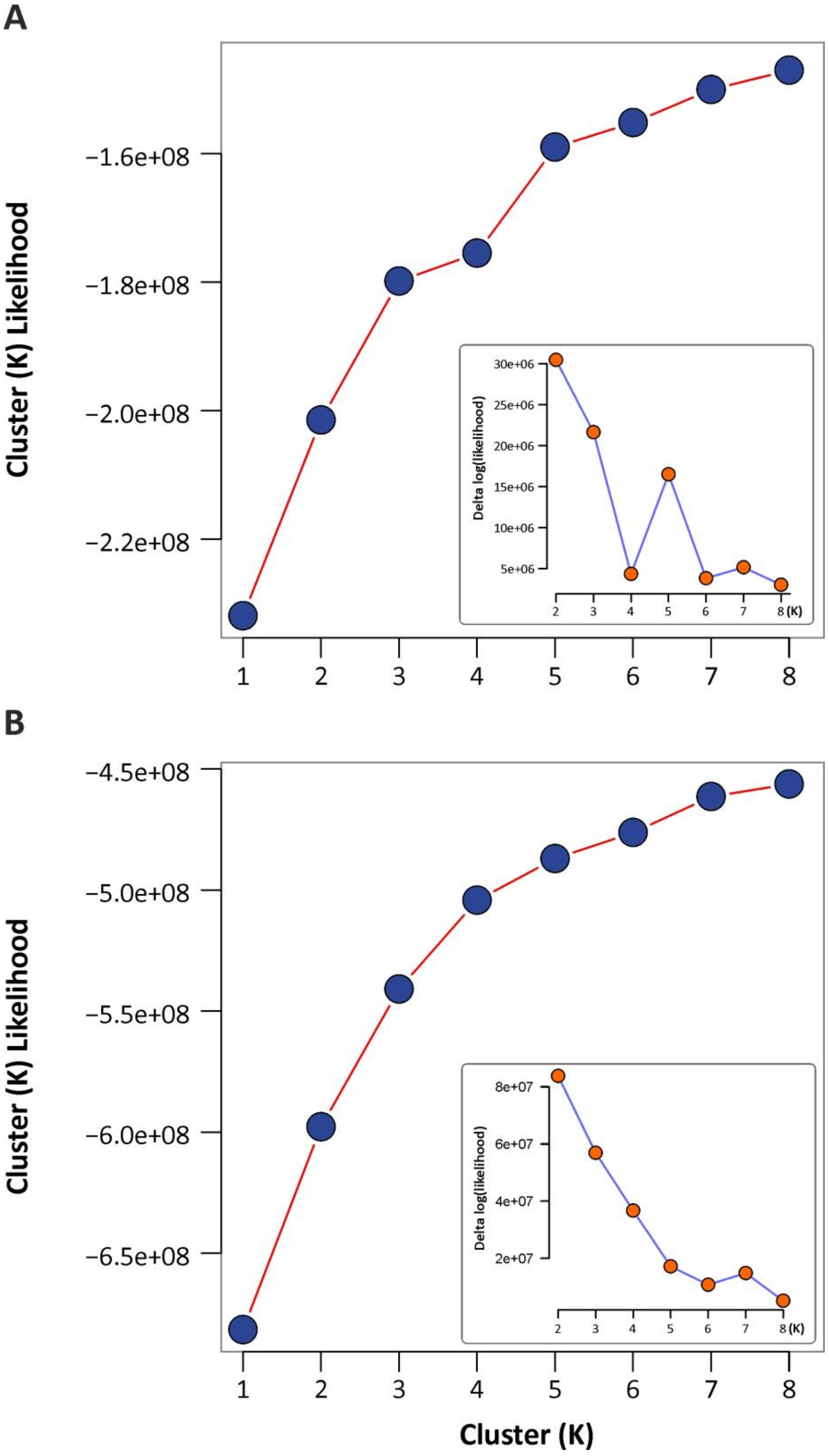

Figure S3. Extended results of D-statistic analyses for the Saqqara date palm leaf amongst date palm populations and closely related species (*P. atlantica*, *P. sylvestris* and *P. theophrasti*), with *P. reclinata* fixed as the outgroup. The outcome of all possible permutations between individuals analysed are provided on Tables S4, S5.

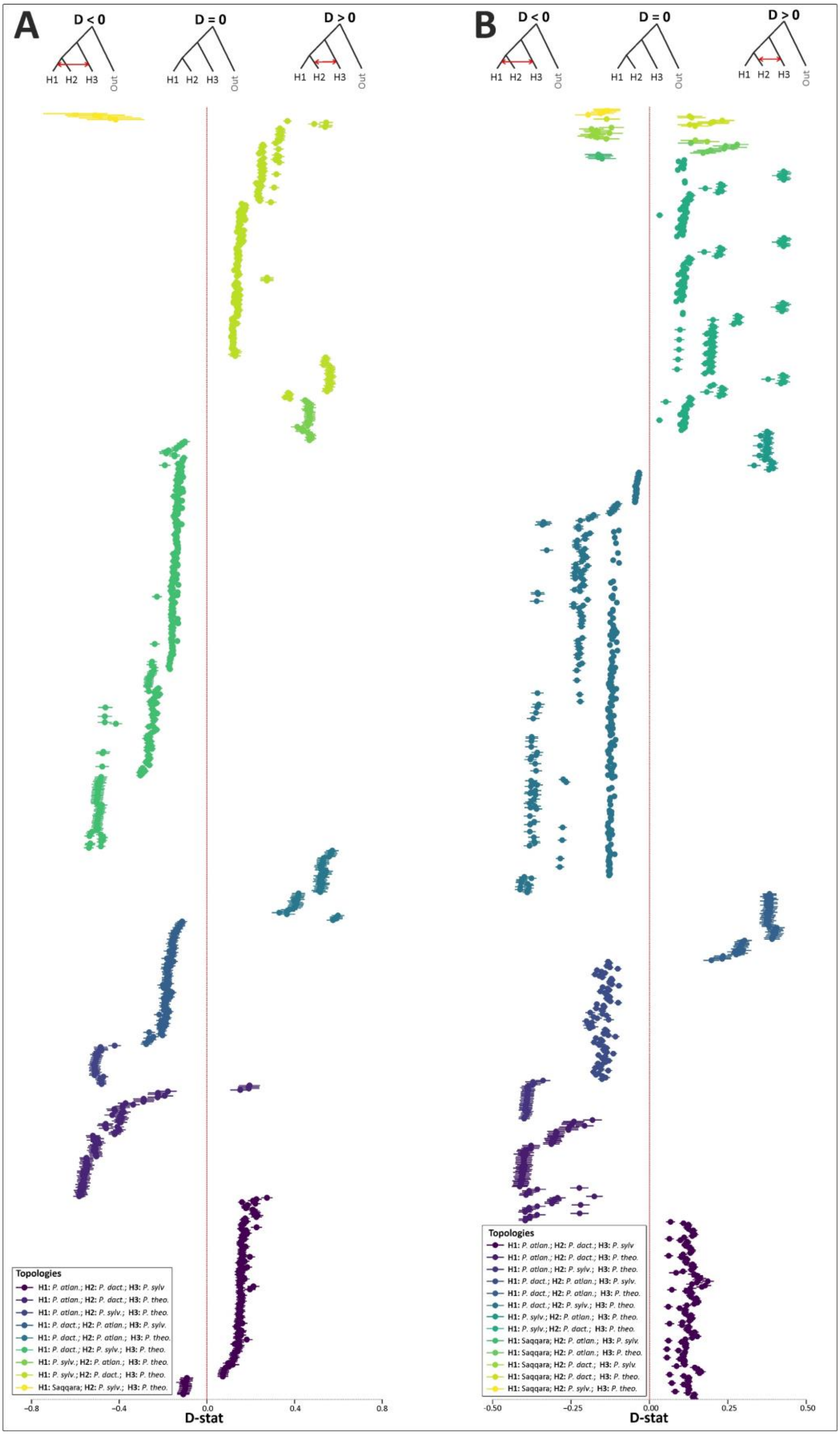

Figure S4. Calibrated phylogeny of *Phoenix* inferred whole plastid genome alignments, a log-normal relaxed molecular clock and a Bayesian Multispecies coalescence tree model. (Inset) Posterior age distribution of the MRCA of *Phoenix* and its corresponding arithmetic mean (dashed line).

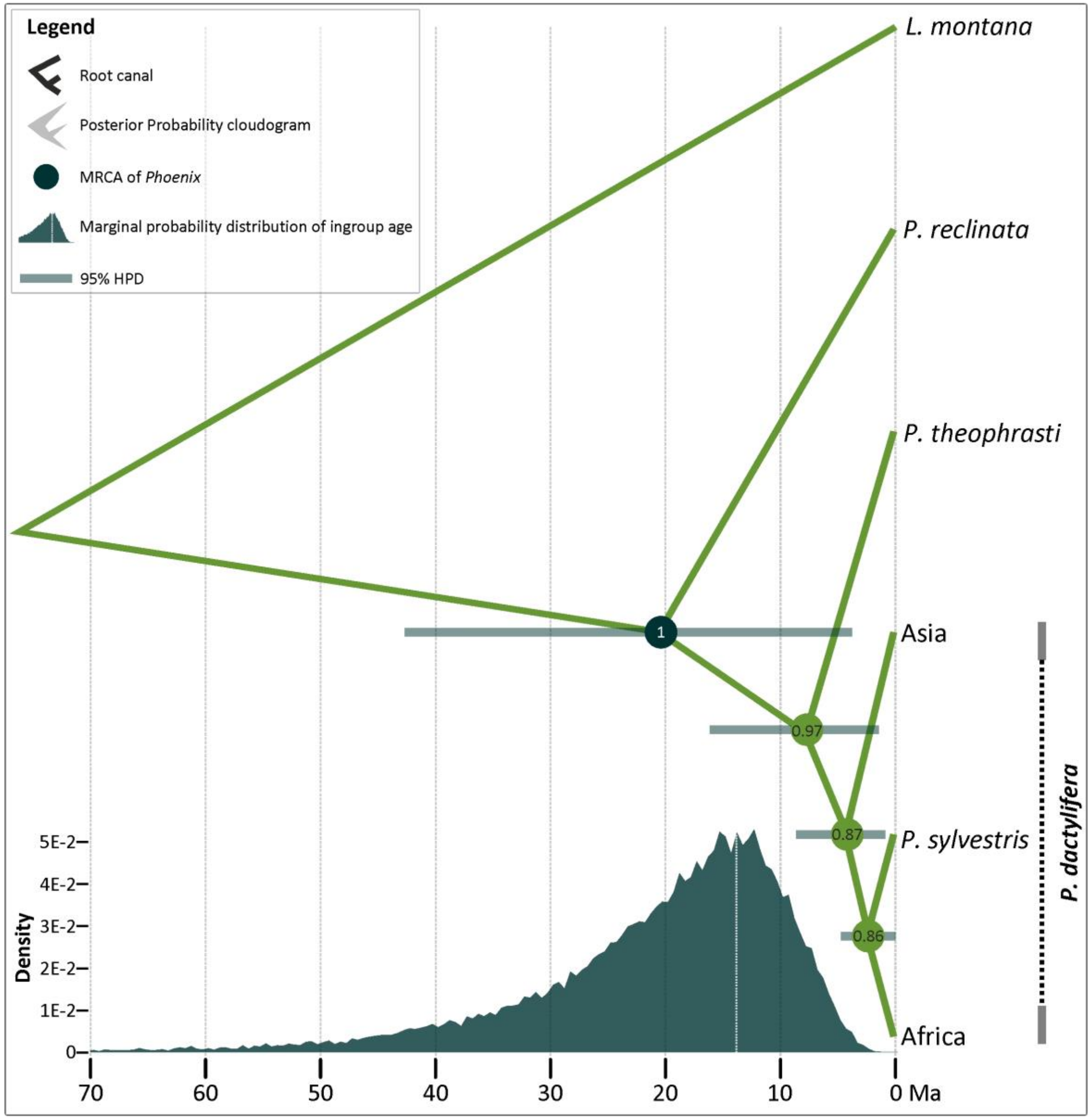

Figure S5. Multispecies coalescence phylogeny of *Phoenix* inferred from 1,143 Maximum Likelihood trees derived from 10,000 bp nuclear genome-wide regions. The numbers at nodes represent the quartet supports (i.e., the percentage of quartets in gene trees that are congruent with the species tree).

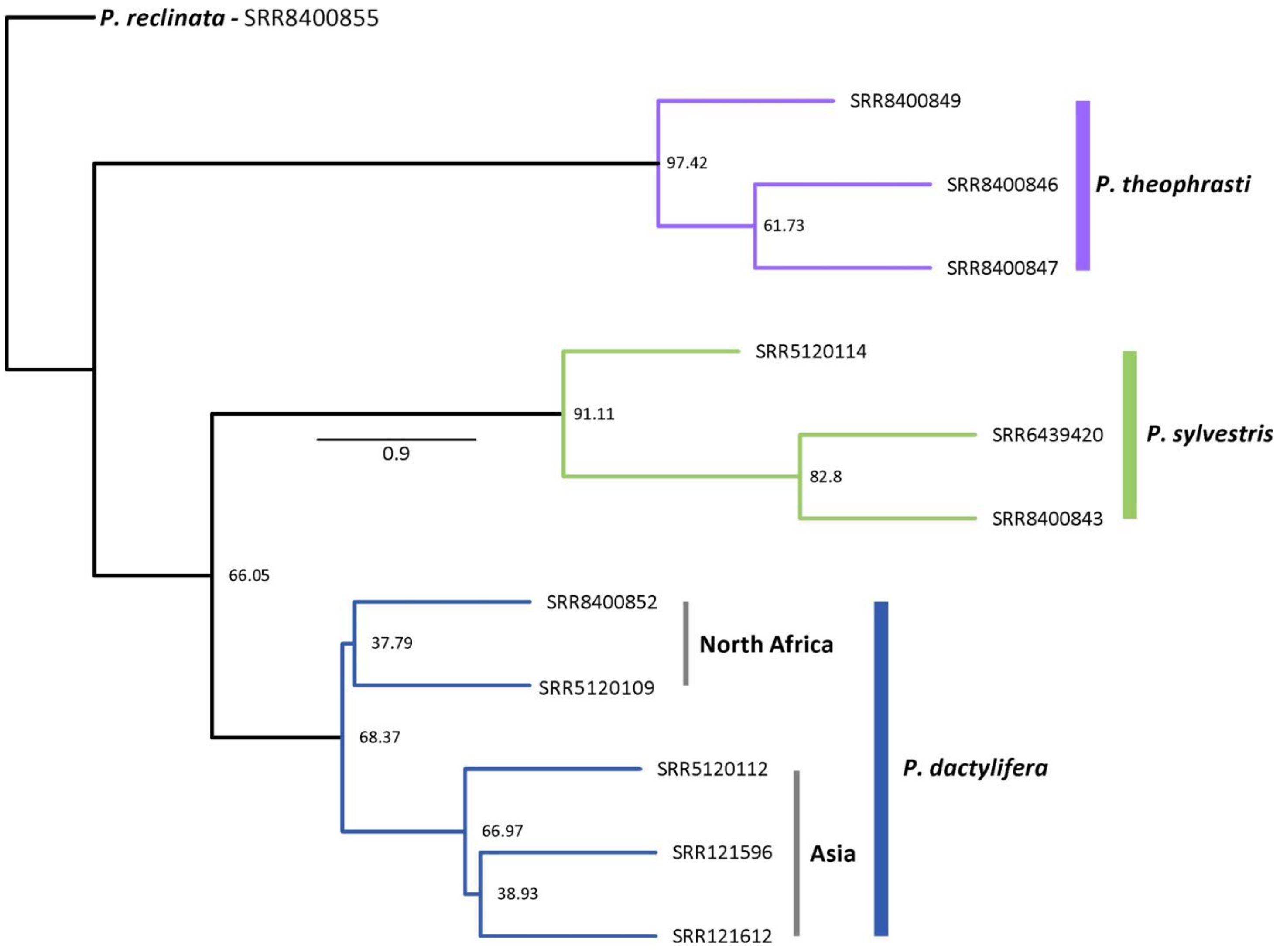

Figure S6. Tree quartets across 18 nuclear scaffolds as inferred in DiscoVista. The topology of possible quartets derived from the true species tree (bottom right) are color coded on the right, and their corresponding relative frequencies are provided on the left.

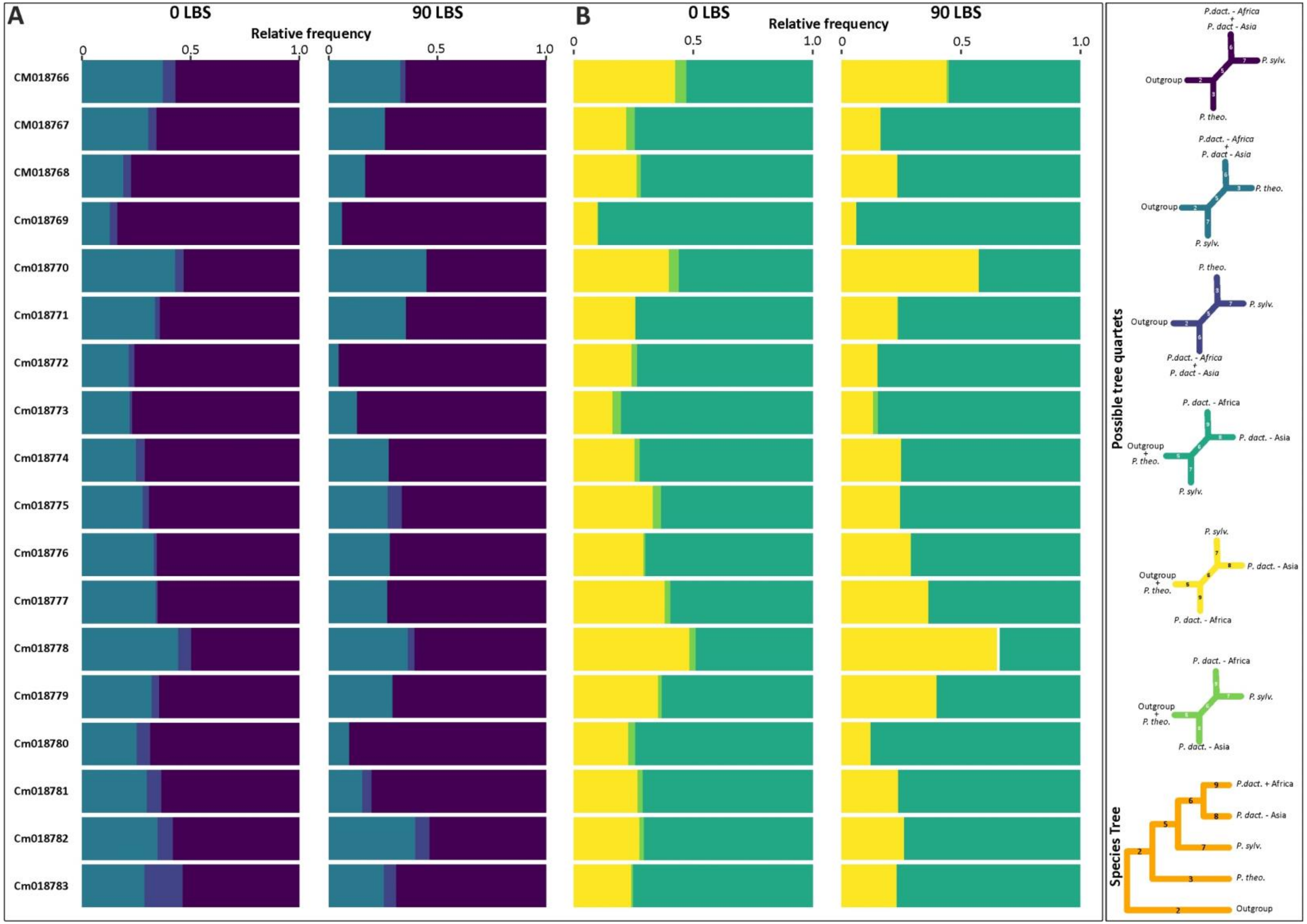

Supplementary Tables

Table S1. Number of raw and retained reads for mapping to genomic scaffolds of the reference (representing ~20% of the nuclear genome of *P. dactylifera*), and their corresponding retained nucleotides. The estimated coverage attained per accession and number of unique hits are also provided.

Table S2. Voucher information of individuals sampled in this study. SRA accession numbers for newly generated and datamined read sequence data are provided, together with detailed information on the provenance of the individual and cultivar name.

Table S3. Summary statistics of plastid and nuclear alignments employed to conduct Maximum Likelihood, NeighborNet and Bayesian Multispecies coalescence analyses. The table contains 1245 rows, and is freely available at https://github.com/siriusb-nox/Archaeogenomics-of-a-2-100-year-old-Egyptian-leaf-provides-a-new-timestamp-on-date-palm-domesticati

Table S4. Summary statistics of D-statistic tests conducted on modern individuals of date palms, their known closely related species and the ancient Saqqara date palm leaf using as a reference a highly fragmented genome reference (GCA_000413155.1). The table contains 3150 rows and nine columns, and is freely available for download at https://github.com/siriusb-nox/Archaeogenomics-of-a-2-100-year-old-Egyptian-leaf-provides-a-new-timestamp-on-date-palm-domesticati

Table S5. Summary statistics of ABBA/BABA introgression tests conducted on modern individuals of date palms, their known closely related species and the ancient Saqqara date palm leaf using as a reference a contiguous genome reference (GCA_009389715.1). The table contains 3372 rows and nine columns, and is freely available for download at https://github.com/siriusb-nox/Archaeogenomics-of-a-2-100-year-old-Egyptian-leaf-provides-a-new-timestamp-on-date-palm-domesticati

Table S6. Summary statistics of ABBA/BABA introgression tests conducted on modern populations of date palms, their known closely related species and the ancient Saqqara date palm leaf using as references the highly fragmented and contiguous genome assemblies GCA_000413155.1 and GCA_009389715.1, respectively. In these analyses, both polymorphic and non-polymorphic sites in the outgroup were considered. Statistically supported introgressive relationships exclusively involving modern populations are highlighted in blue.

Table S7. Summary statistics of ABBA/BABA introgression tests conducted on modern populations of date palms, their known closely related species and the ancient Saqqara date palm leaf using as references the highly fragmented and contiguous genome assemblies GCA_000413155.1 and GCA_009389715.1, respectively. In this analysis, only non-polymorphic sites in the outgroup were considered. Statistically supported introgressive relationships exclusively involving modern populations are highlighted in blue.

Table S8. Summary statistics of absolute age estimation analyses conducted on nuclear and plastid scaffolds**.** PP denotes posterior probabilities. The table contains 175 rows and nine columns, and is freely available for download at https://github.com/siriusb-

nox/Archaeogenomics-of-a-2-100-year-old-Egyptian-leaf-provides-a-new-timestamp-on-date-palm-domesticati

Table S9. Topology frequencies estimated across 18 nuclear scaffolds.

Table S10. Minimum distances between pair of terminals and their significance (raw and Bonferroni adjusted P-values) produced by JML. Significant introgressive relationships (P < 0.05) are highlighted in bold.

Table S1

Individuals	SRA - accession number	No. raw reads[3]	Nuclear reference genome GCA_000413155.1				Nuclear reference genome GCA_009389715.1				Plastid reference genome NC013991.1			
			No. retained reads	No. retained nucleotides	Estimated coverage(X)[1]	Unique hits	No. retained reads	No. retained nucleotides	Estimated coverage(X)[1]	Unique hits	No. retained reads	No. retained nucleotides	Estimated coverage(X)[1]	Unique hits
Saqqara leaf[2]	26796 - SRA	39714855	162901	5526138	0.003	141616	16466010	755175342	0.004057077	156236	18800249	852890081	1.82	89792
Phoenix dactylifera	SRR106852	20000000	31070020	3039671056	9.6	14638118	31070020	3042442034	6.18	24404988	3102681	305551432	111.35	178218
Phoenix dactylifera	SRR121596	20000000	19838543	1231803523	2.81	6742702	19838543	1231803523	2.50	15511640	1987433	123281514	25.94	65781
Phoenix dactylifera	SRR121604	20000000	19615729	1504260087	3.29	6296046	19615729	1504260087	2.97	14842767	1951294	143204817	31.01	64605
Phoenix dactylifera	SRR121607	20000000	19198513	1378633785	3.04	6217758	19198513	1378633785	2.73	14573726	1936467	145811777	31.11	64132
Phoenix dactylifera	SRR121612	20000000	19797898	797195745	1.66	6159719	19797898	797195745	1.52	14533891	1978407	79695386	20.90	81891
Phoenix atlantica	SRR5120109	20000000	36776424	3566681180	11.3	16894245	36776424	3698611431	7.36	28452516	1906380	194287470	46.80	73325
Phoenix dactylifera	SRR5120110	20000000	36505359	3844356688	12.22	17384462	36505359	3852297580	7.91	29106586	1830395	193967026	39.90	60013
Phoenix dactylifera	SRR5120111	20000000	37069871	3545012030	11.62	17492097	37069871	3701052687	7.54	29316817	1885502	186878861	42.41	68388
Phoenix dactylifera	SRR5120112	20000000	37056050	3700304914	11.62	17454900	37056050	3706240502	7.59	29489481	1928963	196111064	45.59	71609
Phoenix dactylifera	SRR5120113	20000000	30212522	3662418910	11.14	13822774	30212522	3665246964	7.23	23195342	1509515	183696672	30.57	39173
Phoenix sylvestris	SRR5120114	20000000	37532609	3868119393	12.24	17786396	37532609	3880569821	7.91	29761580	1878559	195191263	32.36	49730
Phoenix dactylifera	SRR5120115	20000000	32739781	3658971109	11.71	15680109	32739781	3657544258	7.55	26203131	1697699	191288282	33.18	46879
Phoenix canariensis	SRR6439394	20000000	31556641	5289816013	10.33	9107236	31556641	5289816013	7.77	17854936	3099051	522332572	450.94	420933
Phoenix canariensis	SRR6439417	20000000	35031831	3756588201	11.28	15835780	35031831	3755547658	7.24	26275843	3553845	378152224	206.67	307053
Phoenix sylvestris	SRR6439420	20000000	34780744	3632290676	11.46	16249169	34780744	3678910137	7.44	27321054	3518616	368217751	158.75	239508
Phoenix sylvestris	SRR8400836	20000000	31774533	3232222077	9.61	14175411	31774533	3232215832	6.51	24868194	3212667	325639702	49.77	78244
Phoenix sylvestris	SRR8400837	20000000	35731065	3482736313	9.91	15278669	35731065	3482734725	6.91	27588656	3594700	349594193	102.50	168317
Phoenix sylvestris	SRR8400841	20000000	29538517	3069049399	8.97	12969558	29538517	3069028025	6.11	22843816	2983383	309004551	70.62	107862
Phoenix sylvestris	SRR8400842	20000000	34079362	3386887738	9.95	15030562	34079362	3386865946	6.78	26534235	3433998	340263831	53.18	84982
Phoenix sylvestris	SRR8400843	20000000	29992883	3067307079	9.16	13454670	29992883	3067301779	6.16	23391530	3037703	309515461	41.79	64760
Phoenix atlantica	SRR8400845	20000000	35988537	3447397687	10.23	16054094	35988537	3447398344	6.90	27981572	3581874	343604455	56.09	93106
Phoenix theophrasti	SRR8400846	20000000	33522650	3686406335	9.92	13530570	33522650	3686174682	6.92	24445554	3362989	369706858	150.32	219029
Phoenix theophrasti	SRR8400847	20000000	34034851	3709357487	10.01	13778296	34034851	3709153760	7.00	24961314	3413330	371990883	132.68	195108
Phoenix theophrasti	SRR8400849	20000000	32362379	3579490581	10.15	13752262	32362379	3578569589	7.00	24549010	3269598	360232131	72.24	104822
Phoenix dactylifera	SRR8400852	20000000	36174487	3525310483	9.95	15308853	36174487	3525300103	6.88	27425519	3602457	351838427	75.00	123176
Phoenix reclinata	SRR8400855	20000000	37163656	3579249166	10.25	15990169	37163656	3579240233	6.92	27956758	3715296	358003100	86.87	144489
Phoenix canariensis	SRR8400858	20000000	36863309	3534810726	10.03	15725507	36863309	3534811394	6.65	26989431	3709622	354967742	118.84	198397
Phoenix theophrasti	SRR8400865	20000000	35053705	3734830941	10.19	14342669	35053705	3733775757	7.08	25803338	3530027	375209833	97.12	145933
Phoenix dactylifera	SRR974758	20000000	39551815	3512961021	9.86	16966099	39551815	3512961021	7.08	31437142	3956435	350749040	49.31	126340
Phoenix dactylifera	SRR974793	20000000	39536947	3513471886	9.9	17026521	39536947	3513471886	7.08	31449887	3954913	350713510	40.87	74606
Phoenix dactylifera	SRR974794	20000000	39429272	3535432472	10.05	17130732	39429272	3535432472	7.16	31472720	3944361	354135748	54.77	98585
Phoenix dactylifera	SRR974795	20000000	39329305	3522692720	9.98	17017292	39329305	3522692720	7.12	31324798	3935595	352820111	65.73	118457
Phoenix dactylifera	SRR974796	20000000	39423381	3533849702	9.99	17026173	39423381	3533849702	7.11	31263471	3943149	353963579	50.34	90646
Phoenix dactylifera	SRR974797	20000000	39401122	3533817817	10	17037479	39401122	3533817817	7.094833777	31191366	3941913	353305389	48.43	87368

[1]From unique hits

[2]Target of 198 contigs, or 149010247 bases

[3]Single-end reads for libaries of the Saqqara date leaf, SRR121596, SRR121604, SRR121607, SRR121612

Table S2

Species	Sex	SRA - accession number	Place of origin	Locality	Notes	Population
Licuala montana	Unknown	PL34 - SRA	Unknown	Unknown	Outgroup	-
Phoenix atlantica	Unknown	SRR5120109	Africa	Cape Verde	Wild individual	P.atlantica
Phoenix atlantica	Female	SRR8400845	Africa	Maio	Wild individual	P.atlantica
Phoenix canariensis	Male	SRR6439394	Unknown	USA	Collected in a botanical garden	-
Phoenix canariensis	Female	SRR6439417	Unknown	USA	Collected in a botanical garden	-
Phoenix canariensis	Male	SRR8400858	Europe	Italy	Collected in a botanical garden	-
Phoenix dactylifera	Unknown	26796 - SRA	Africa	Saqqara	Ancient Saqqara date palm	Saqqara
Phoenix dactylifera	Female	SRR106852	Unknown	Unknown	Khalass cultivar	P. dactylifera
Phoenix dactylifera	Unknown	SRR121596	Asia	SaudAra	Khalas cultivar	P. dactylifera
Phoenix dactylifera	Female	SRR121604	Africa	Morocco	Medjool cultivar	P. dactylifera
Phoenix dactylifera	Unknown	SRR121607	Africa	Unknown	Deglet Noor cultivar	P. dactylifera
Phoenix dactylifera	Unknown	SRR121612	Asia	Unknown	Al-Rijal cultivar	P. dactylifera
Phoenix dactylifera	Unknown	SRR5120110	Africa	Egypt	Siwi cultivar	P. dactylifera
Phoenix dactylifera	Unknown	SRR5120111	Asia	Oman	Wild individual	P. dactylifera
Phoenix dactylifera	Unknown	SRR5120112	Asia	Oman	Jabri cultivar	P. dactylifera
Phoenix dactylifera	Unknown	SRR5120113	Asia	Oman	Wild individual	P. dactylifera
Phoenix dactylifera	Unknown	SRR5120115	Asia	Oman	Wild individual	P. dactylifera
Phoenix dactylifera	Female	SRR8400852	Africa	Morocco	Khalte cultivar	P. dactylifera
Phoenix dactylifera	Unknown	SRR974758	Asia	Saudi Arabia	Perny cultivar	P. dactylifera
Phoenix dactylifera	Unknown	SRR974793	Asia	Saudi Arabia	Dekhaini cultivar	P. dactylifera
Phoenix dactylifera	Unknown	SRR974794	Asia	Saudi Arabia	SukkariatQa cultivar	P. dactylifera
Phoenix dactylifera	Unknown	SRR974795	Asia	Saudi Arabia	Rabia cultivar	P. dactylifera
Phoenix dactylifera	Unknown	SRR974796	Asia	Saudi Arabia	Shalaby cultivar	P. dactylifera
Phoenix dactylifera	Unknown	SRR974797	Asia	Saudi Arabia	Moshwaq Al-Riyad	P. dactylifera
Phoenix reclinata	Unknown	SRR8400855	Africa	USA	Collected in a botanical garden	P. reclinata
Phoenix sylvestris	Unknown	SRR5120114	Asia	India	Wild individual	P. sylvestris
Phoenix sylvestris	Female	SRR6439420	Asia	USA	Collected in a botanical garden	P. sylvestris
Phoenix sylvestris	Unknown	SRR8400836	Europe	Spain	Collected in a botanical garden	P. sylvestris
Phoenix sylvestris	Unknown	SRR8400837	Unknown	Unknown	-	P. sylvestris
Phoenix sylvestris	Unknown	SRR8400841	Asia	USA	Collected in a botanical garden	P. sylvestris
Phoenix sylvestris	Unknown	SRR8400842	Asia	USA	Collected in a botanical garden	P. sylvestris
Phoenix sylvestris	Male	SRR8400843	Asia	USA	Collected in a botanical garden	P. sylvestris
Phoenix theophrasti	Male	SRR8400846	Greece	Drapano	Wild individual	P. theophrasti
Phoenix theophrasti	Female	SRR8400847	Greece	Drapano	Wild individual	P. theophrasti
Phoenix theophrasti	Unknown	SRR8400849	Greece	Almyros	Wild individual	P. theophrasti
Phoenix theophrasti	Unknown	SRR8400865	Greece	Maridak	Wild individual	P. theophrasti

Table S6

Reference genome GCA_000413155.1										
H1[a]	H2	H3	H4	D	Z	p-value	number of ABBA	number of BABA	Total mumber of sites[b]	number of Blocks
Saqqara	Pdact	Psylv	Precli	-0.0209	-1.06017	0.289069	207.4114	216.265	423.676431	194
Saqqara	Pdact	Ptheo	Precli	0.011419	0.59606	0.551135	193.3996	189.0325	382.432115	192
Saqqara	Patlan	Psylv	Precli	-0.06622	-3.13113	0.001741	215.7791	246.3831	462.162213	191
Saqqara	Patlan	Ptheo	Precli	0.119498	5.523541	0	252.0099	198.2098	450.219678	190
Saqqara	Psylv	Ptheo	Precli	-0.05391	-2.29486	0.021741	209.8369	233.7521	443.588983	191
Pdact	Patlan	Psylv	Precli	-0.08461	-10.8521	0	48890.03	57927.48	106817.5171	198
Pdact	Patlan	Ptheo	Precli	0.262586	17.13452	0	71961.45	42029.13	113990.5749	198
Pdact	Psylv	Ptheo	Precli	-0.17642	-25.5788	0	51186.17	73115.09	124301.2615	198
Patlan	Psylv	Ptheo	Precli	-0.35284	-28.5593	0	47658.63	99627	147285.6288	197

[b]Total No. of sites evaluated for permutations involving the Saqqara ancient genome: 2162.08 (average= 432.415884)

Reference genome GCA_009389715.1										
H1[a]	H2	H3	H4	D	Z	p-value	number of ABBA	number of BABA	Total mumber of sites[b]	number of Blocks
Saqqara	Pdact	Psylv	Precli	-0.07473	-3.27382	0.001061	277.2991	322.0905	599.389583	84
Saqqara	Pdact	Ptheo	Precli	0.023902	1.048354	0.294476	275.8091	262.9319	538.741018	84
Saqqara	Patlan	Psylv	Precli	-0.11742	-4.20996	0.000026	302.3736	382.8322	685.205767	84
Saqqara	Patlan	Ptheo	Precli	0.202933	7.815479	0	430.1243	285.0015	715.125802	84
Saqqara	Psylv	Ptheo	Precli	-0.12568	-3.90997	0.000092	365.6533	470.7707	836.424047	84
Pdact	Patlan	Psylv	Precli	-0.10697	-11.3274	0	104668	129743.3	234411.2805	84
Pdact	Patlan	Ptheo	Precli	0.274172	13.37589	0	161093	91766.17	252859.1701	84
Pdact	Psylv	Ptheo	Precli	-0.21301	-24.7303	0	111525.2	171895.8	283420.9415	84
Patlan	Psylv	Ptheo	Precli	-0.38139	-24.8669	0	104776.2	233972.5	338748.6974	84

[b]Total No. of sites evaluated for permutations involving the Saqqara ancient genome: 3374.886217 (average= 674.9772434)

[a]Patlan: *P. atlantica* ; Ptheo: *P. theophrasti* ; Psyvl: *P. sylvestris* ; Pdact: *P. dactylifera*

Supported D-statistics suggesting introgression between the Saqqara leaf genome, *P. theophrasti* and *P. sylvestris*

Supported D-statistics suggesting introgression between the *P.dactylifera, P. sylvestris* and *P. theophrasti*

Table S7

Reference genome GCA_000413155.1										
H1[a]	H2	H3	H4	D	Z	p-value	number ABBA	number BABA	Total mumber of sites[b]	number of Blocks
Saqqara	Pdact	Psylv	Precli	0.032294	0.737717	0.460687	61.01021	57.193	118.203208	193
Saqqara	Pdact	Ptheo	Precli	0.076138	1.628711	0.103374	48.89833	41.97906	90.877396	191
Saqqara	Patlan	Psylv	Precli	-0.07933	-1.68605	0.091786	64.4214	75.52325	139.944651	190
Saqqara	Patlan	Ptheo	Precli	0.335279	7.682932	0	91.2417	45.42144	136.663133	189
Saqqara	Psylv	Ptheo	Precli	-0.14626	-2.8548	0.004306	59.6628	80.10532	139.76812	191
Pdact	Patlan	Psylv	Precli	-0.11119	-11.0763	0	34142.24	42684.59	76826.83095	198
Pdact	Patlan	Ptheo	Precli	0.339534	18.72564	0	56624.08	27918.87	84542.95766	197
Pdact	Psylv	Ptheo	Precli	-0.22489	-26.2835	0	35485.72	56077.29	91563.01425	197
Patlan	Psylv	Ptheo	Precli	-0.43406	-31.3139	0	32204.91	81605.5	113810.4108	197

[b]Total No. of sites evaluated for permutations involving the Saqqara ancient genome: 625.456508 (average=125.0913016)

Reference genome GCA_009389715.1										
H1[a]	H2	H3	H4	D	Z	p-value	number of ABBA	number of BABA	Total mumber of sites[b]	number of Blocks
Saqqara	Pdact	Psylv	Precli	-0.01344	-0.40904	0.682514	168.3591	172.9461	341.305183	84
Saqqara	Pdact	Ptheo	Precli	0.101126	2.734258	0.006252	159.0302	129.8199	288.850088	84
Saqqara	Patlan	Psylv	Precli	-0.05225	-1.23309	0.217541	182.8642	203.026	385.890162	84
Saqqara	Patlan	Ptheo	Precli	0.344698	10.00946	0	279.9476	136.4248	416.372439	84
Saqqara	Psylv	Ptheo	Precli	-0.12464	-2.83039	0.004649	220.7346	283.5946	504.329183	84
Pdact	Patlan	Psylv	Precli	-0.1256	-11.0738	0	78700.71	101309.6	180010.312	84
Pdact	Patlan	Ptheo	Precli	0.328078	14.38501	0	132116.9	66842.66	198959.5924	84
Pdact	Psylv	Ptheo	Precli	-0.25064	-24.653	0	82591.13	137839	220430.1338	84
Patlan	Psylv	Ptheo	Precli	-0.44018	-26.8196	0	76372.93	196473.8	272846.6866	84

[b]Total No. of sites evaluated for permutations involving the Saqqara ancient genome: 1936.747055 (average=387.349411)

[a]Patlan: *P. atlantica* ; Ptheo: *P. theophrasti* ; Psyvl: *P. sylvestris* ; Pdact: *P. dactylifera*

Supported D-statistics suggesting introgression between the Saqqara leaf genome, *P. theophrasti* and *P. sylvestris*

Supported D-statistics suggesting introgression between the *P.dactylifera, P. sylvestris* and *P. theophrasti*

Table S9

	T1[1]	T2	T3	T4	T5	T6	T7	T8	T9	T10	T11	T12
CM018766	0.026315789	0.247368421	0.005263158	0.026315789	0.005263158	0.005263158	0.026315789	0.031578947	0.057894737	0.010526316	0.094736842	0.463157895
CM018767	0.037974684	0.101265823	0	0	0	0	0.037974684	0.037974684	0.088607595	0	0.101265823	0.594936709
CM018768	0.034883721	0.197674419	0	0.023255814	0	0	0.011627907	0.034883721	0.023255814	0	0.046511628	0.627906977
CM018769	0.016666667	0.016666667	0	0	0	0	0	0.05	0.016666667	0	0.033333333	0.866666667
CM018770	0.022222222	0.222222222	0	0	0	0	0.044444444	0.044444444	0.066666667	0	0.133333333	0.466666667
CM018771	0	0.181818182	0	0.018181818	0	0	0	0.054545455	0.036363636	0	0.163636364	0.545454545
CM018772	0	0.216666667	0	0	0	0	0	0.083333333	0.083333333	0	0.05	0.566666667
CM018773	0	0.125	0	0.011363636	0.011363636	0	0	0.022727273	0.079545455	0	0.113636364	0.636363636
CM018774	0.055555556	0.111111111	0	0	0	0	0.027777778	0.055555556	0.013888889	0	0.125	0.611111111
CM018775	0.029411765	0.176470588	0	0	0	0	0.029411765	0.058823529	0	0	0.117647059	0.588235294
CM018776	0.013888889	0.111111111	0	0	0	0	0.013888889	0.013888889	0.083333333	0	0.097222222	0.666666667
CM018777	0	0.225	0	0.025	0	0	0.05	0.1	0.025	0	0.075	0.5
CM018778	0.046511628	0.279069767	0	0.093023256	0	0	0	0	0.139534884	0	0.046511628	0.395348837
CM018779	0.030769231	0.246153846	0	0	0	0	0.015384615	0.030769231	0.153846154	0	0.030769231	0.492307692
CM018780	0.03030303	0.212121212	0	0	0	0	0	0.060606061	0.090909091	0	0.03030303	0.575757576
CM018781	0.026315789	0.105263158	0	0.026315789	0	0	0.052631579	0.026315789	0	0	0.026315789	0.736842105
CM018782	0.039215686	0.137254902	0	0	0	0.019607843	0	0.039215686	0.039215686	0	0.039215686	0.68627451
CM018783	0.1875	0.0625	0	0	0	0	0	0.0625	0	0	0.0625	0.625

[1]**Key**

T1: (R,((T,S),(As,Af)))
T2: (R,((As,S),(T,Af)))
T3: (R,((S,Af),(As,T)))
T4: (R,(As,(S,(Af,T))))
T5: (R,(As,(T,(Af,S))))
T6: (R,(Af,(T,(As,S))))
T7: (R,(T,(As,(Af,S))))
T8: (R,(T,(Af,(S,As))))
T9: (R,(S,(As,(Af,T))))
T10: (R,(S,(Af,(As,T))))
T11: (R,(S,(T,(As,Af))))
T12: (R,(T,(S,(As,Af))))

R: P. reclinata, T: P. theophrasti, S: P. sylvestris, As: Asian P. dactylifera, Af: North African P. dactylifera

Table S10

Comparison	Minimum distance	Adjuste P-value
Ptheo-Precl	0.00913103	0.11879946
Pdact-Precl	0.00908265	0.14489928
Pdact-Ptheo	0.00792820	0.19259820
Psylv-Precl	0.00928451	0.14399928
Psylv-Ptheo	0.00851401	0.20429820
Psylv-Pdact	0.00665773	0.14219928
Pdact_Africa-Precl	0.00909305	0.13229928
Pdact_Africa-Ptheo	0.00575404	0.12149946
Pdact_Africa-Pdact	0.00511048	0.27179820
Pdact_Africa-Psylv	0.00715718	0.18179820
Ptheo-Precl	0.00886990	0.87422400
Pdact-Precl	0.00829872	1.00000000
Pdact-Ptheo	0.00602298	0.42425460
Psylv-Precl	0.00886862	1.00000000
Psylv-Ptheo	0.00730621	0.68137920
Psylv-Pdact	0.00522896	0.73280520
Pdact_Africa-Precl	0.00843063	1.00000000
Pdact_Africa-Ptheo	0.00562213	0.37283040
Pdact_Africa-Pdact	0.00313251	1.00000000
Pdact_Africa-Psylv	0.00567976	0.96421680
Ptheo-Precl	0.00868859	1.00000000
Pdact-Precl	0.00820843	1.00000000
Pdact-Ptheo	0.00697693	1.00000000
Psylv-Precl	0.00870620	1.00000000
Psylv-Ptheo	0.00786915	1.00000000
Psylv-Pdact	0.00555055	1.00000000
Pdact_Africa-Precl	0.00814269	1.00000000
Pdact_Africa-Ptheo	0.00596144	1.00000000
Pdact_Africa-Pdact	0.00290089	1.00000000
Pdact_Africa-Psylv	0.00586987	1.00000000
Pdact_Africa-Pdact	0.00294938	**0.02699874**
Pdact_Africa-Pdact	0.00299693	**0.04499784**
Pdact_Africa-Psylv	0.00541308	**0.02699874**
Pdact_Africa-Psylv	0.00672215	**0.04499784**
Pdact_Africa-Psylv	0.00592402	**0.01799910**
Pdact_Africa-Ptheo	0.00616765	**0.02699874**
Ptheo-Precl	0.00873781	0.14999166
Pdact-Precl	0.00847836	0.28998360
Pdact_Africa-Ptheo	0.00447833	**0.02999826**
Psylv-Precl	0.00887092	0.37997820
Psylv-Ptheo	0.00699837	0.10999386
Psylv-Pdact	0.00557478	0.19998900
Pdact_Africa-Precl	0.00845354	0.27998460
Pdact_Africa-Ptheo	0.00620198	**0.02571246**
Pdact_Africa-Pdact	0.00396619	0.20998800
Pdact_Africa-Psylv	0.00613204	0.23998680
Ptheo-Precl	0.00828994	0.16713090
Pdact-Precl	0.00830643	0.17998722
Pdact-Ptheo	0.00573060	0.65566800
Psylv-Precl	0.00870215	0.24426900
Psylv-Ptheo	0.00674737	0.52710480
Psylv-Pdact	0.00531839	0.23141160
Pdact_Africa-Precl	0.00812689	0.11570598
Pdact_Africa-Ptheo	0.00501977	0.46282320
Pdact_Africa-Pdact	0.00312911	0.74566080
Pdact_Africa-Psylv	0.00557487	0.28283760
Ptheo-Precl	0.00838962	0.08999352
Pdact-Precl	0.00826084	0.07713738
Pdact_Africa-Ptheo	0.00497462	**0.04499784**
Psylv-Precl	0.00862855	0.07713738
Pdact_Africa-Ptheo	0.00587240	**0.01799910**
Psylv-Pdact	0.00530049	0.10284984
Pdact_Africa-Precl	0.00811002	0.07713738
Pdact_Africa-Ptheo	0.00599766	**0.02699874**
Pdact_Africa-Pdact	0.00264177	0.10284984
Pdact_Africa-Psylv	0.00535811	0.12856230
Ptheo-Precl	0.00825895	0.82282860
Pdact-Precl	0.00749105	1.00000000
Pdact-Ptheo	0.00636670	0.75854520
Psylv-Precl	0.00827385	0.98353620
Psylv-Ptheo	0.00746469	1.00000000
Psylv-Pdact	0.00512087	1.00000000
Pdact_Africa-Precl	0.00758503	1.00000000
Pdact_Africa-Ptheo	0.00604923	0.70068960

Pdact_Africa-Pdact	0.00249052	1.00000000
Pdact_Africa-Psylv	0.00529966	1.00000000
Ptheo-Precl	0.00883271	0.33426180
Pdact-Precl	0.00836930	0.28283760
Pdact-Ptheo	0.00683989	0.47568060
Psylv-Precl	0.00910825	0.39854340
Psylv-Ptheo	0.00790171	0.38568600
Psylv-Pdact	0.00546217	0.23141160
Pdact_Africa-Precl	0.00846393	0.38568600
Pdact_Africa-Ptheo	0.00564308	0.39854340
Pdact_Africa-Pdact	0.00326617	1.00000000
Pdact_Africa-Psylv	0.00609258	0.47568060
Ptheo-Precl	0.00873147	0.14141844
Pdact-Precl	0.00828885	0.07713738
Pdact-Ptheo	0.00631180	0.16713090
Psylv-Precl	0.00900590	0.11570598
Psylv-Ptheo	0.00737115	0.28283760
Psylv-Pdact	0.00557115	0.12856230
Pdact_Africa-Precl	0.00831246	0.10284984
Pdact_Africa-Ptheo	0.00457672	0.12856230
Pdact_Africa-Pdact	0.00315443	0.37283040
Pdact_Africa-Psylv	0.00601082	0.17998722
Ptheo-Precl	0.00878094	0.38568600
Pdact-Precl	0.00829581	0.48853620
Pdact-Ptheo	0.00632333	0.20570040
Psylv-Precl	0.00905061	0.83565540
Psylv-Ptheo	0.00772589	0.30854880
Psylv-Pdact	0.00568252	0.41139900
Pdact_Africa-Precl	0.00834864	0.50139360
Pdact_Africa-Ptheo	0.00547123	0.05142492
Pdact_Africa-Pdact	0.00332639	0.79708680
Pdact_Africa-Psylv	0.00618433	0.50139360
Ptheo-Precl	0.00837752	0.08999550
Pdact-Precl	0.00810045	0.08099604
Psylv-Precl	0.00868985	0.15299244
Psylv-Ptheo	0.00684105	0.08999550
Psylv-Pdact	0.00521895	0.16199190
Pdact_Africa-Precl	0.00809037	0.08999550
Pdact_Africa-Pdact	0.00357165	0.18899100
Pdact_Africa-Psylv	0.00576553	0.13499334
Pdact-Ptheo	0.00684411	0.06299694
Pdact-Ptheo	0.00684492	**0.01799910**
Psylv-Ptheo	0.00798870	0.05399730
Pdact-Ptheo	0.00554771	**0.04999716**
Pdact-Ptheo	0.00691199	**0.02571246**
Pdact_Africa-Ptheo	0.00469331	0.13499334
Pdact_Africa-Pdact	0.00444234	0.07199640
Pdact-Ptheo	0.00540786	**0.04499784**
Ptheo-Precl	0.00843063	0.19799100
Pdact-Precl	0.00802516	0.14399280
Pdact-Ptheo	0.00642044	0.90895500
Psylv-Precl	0.00876904	0.26098740
Psylv-Ptheo	0.00756979	0.61196940
Psylv-Pdact	0.00558299	0.26098740
Pdact_Africa-Precl	0.00801269	0.10799460
Pdact_Africa-Ptheo	0.00499818	0.55797300
Pdact_Africa-Pdact	0.00354005	0.71096400
Pdact_Africa-Psylv	0.00598690	0.44097840
Pdact-Ptheo	0.00702016	**0.00899955**
Pdact-Ptheo	0.00748423	**0.03599820**
Psylv-Pdact	0.00528872	**0.02699874**
Psylv-Pdact	0.00582617	**0.01799910**
Psylv-Pdact	0.00591491	**0.04499784**
Psylv-Ptheo	0.00750202	**0.02699874**
Psylv-Ptheo	0.00769825	**0.03856860**
Ptheo-Precl	0.00868070	0.16199190
Pdact-Precl	0.00843537	0.17099154
Pdact-Ptheo	0.00716562	0.35098200
Psylv-Precl	0.00884245	0.17099154
Psylv-Ptheo	0.00784498	0.31498380
Psylv-Pdact	0.00524886	0.20698920
Pdact_Africa-Precl	0.00841920	0.17999100
Pdact_Africa-Ptheo	0.00547800	0.10799460
Pdact_Africa-Pdact	0.00277944	0.25198740
Pdact_Africa-Psylv	0.00557506	0.11699424
Ptheo-Precl	0.00870882	0.37798200

Pdact-Precl	0.00843921	0.36898200
Pdact-Ptheo	0.00580654	0.26998740
Psylv-Precl	0.00916478	0.42297840
Psylv-Ptheo	0.00735482	0.45897660
Psylv-Pdact	0.00547151	0.35098200
Pdact_Africa-Precl	0.00841146	0.39598020
Pdact_Africa-Ptheo	0.00485894	0.13499334
Pdact_Africa-Pdact	0.00308268	0.90895500
Pdact_Africa-Psylv	0.00599091	0.55797300
Ptheo-Precl	0.00872678	0.08099604
Psylv-Ptheo	0.00762441	**0.02699874**
Psylv-Precl	0.00890658	0.07199640
Psylv-Ptheo	0.00817454	0.05399730
Psylv-Pdact	0.00599445	0.07199640
Pdact_Africa-Pdact	0.00316258	0.09899514
Pdact_Africa-Psylv	0.00615819	0.08999550
